# A genetic framework controlling the differentiation of intestinal stem cells during regeneration in *Drosophila*

**DOI:** 10.1371/journal.pgen.1006854

**Published:** 2017-06-29

**Authors:** Zongzhao Zhai, Jean-Philippe Boquete, Bruno Lemaitre

**Affiliations:** Global Health Institute, School of Life Sciences, Ecole Polytechnique Fédérale de Lausanne (EPFL), Station 19, Lausanne, Switzerland; Stanford University School of Medicine, UNITED STATES

## Abstract

The speed of stem cell differentiation has to be properly coupled with self-renewal, both under basal conditions for tissue maintenance and during regeneration for tissue repair. Using the *Drosophila* midgut model, we analyze at the cellular and molecular levels the differentiation program required for robust regeneration. We observe that the intestinal stem cell (ISC) and its differentiating daughter, the enteroblast (EB), form extended cell-cell contacts in regenerating intestines. The contact between progenitors is stabilized by cell adhesion molecules, and can be dynamically remodeled to elicit optimal juxtacrine Notch signaling to determine the speed of progenitor differentiation. Notably, increasing the adhesion property of progenitors by expressing *Connectin* is sufficient to induce rapid progenitor differentiation. We further demonstrate that JAK/STAT signaling, Sox21a and GATAe form a functional relay to orchestrate EB differentiation. Thus, our study provides new insights into the complex and sequential events that are required for rapid differentiation following stem cell division during tissue replenishment.

## Author summary

Adult tissue/organ function is maintained by stem cells. Key question in stem cell biology is how the pool of stem cells can be robustly expanded yet timely contracted through differentiation according to the need of a tissue. Over the last years, the mechanisms underlying stem cell activation have been extensively studied, while the genetic control of progenitor differentiation, especially during regeneration, is still poorly understood. Using the fruit fly *Drosophila* midgut as model, we investigate the cellular changes and the genetic program required for efficient progenitor differentiation during intestinal regeneration. We first detect the presence of extended cell-cell contact between a stem cell and its differentiating daughter in regenerating intestine, compared to homeostatic conditions. The extended cell-cell contact is consolidated by cell adhesion molecules and enhances Notch signaling in the differentiating progenitors leading to their fast differentiation into enterocytes. We further uncover a genetic program, involving the JAK/STAT and Dpp signaling, the Sox21a and GATAe transcription factors, which acts in the differentiating progenitors to instruct their terminal differentiation. Thus, our study presents an integrated view of stem cell differentiation during tissue regeneration and the findings here are likely to apply to mammals.

## Introduction

In metazoans, the digestive tract supports organismal growth and maintenance. Genetic disorders or microbial dysbiosis that prevent the digestion and absorption of nutrients are major causes of morbidity and mortality in humans. In mammals, mature intestinal cells are short-lived and constantly replaced by newborn differentiated cells. This is ensured by the existence of fast-cycling intestinal stem cells (ISCs) [1]. Although ISC division is important, failure in or improper differentiation into mature intestinal cells can equally cause a wide range of disorders that compromise organ function, such as intestinal cancer [2] and microvillus inclusion disease [3].

There is a great extent of similarity in intestinal functions and maintenance between flies and mammals [4]. Over the past decade, research has revealed the extreme plasticity of the *Drosophila* ISCs. For instance, stem cell activity and epithelial renewal can be adjusted in response to i) changes in nutrient availability [5–7], ii) physiological requirements for reproduction [8–10], iii) aging [11–13], iv) intestinal damage or infection [14–16], and v) body injury [17, 18]. Thus, both local and remote signals coordinate ISC activity to ensure intestinal homeostasis.

**Fig 1:**
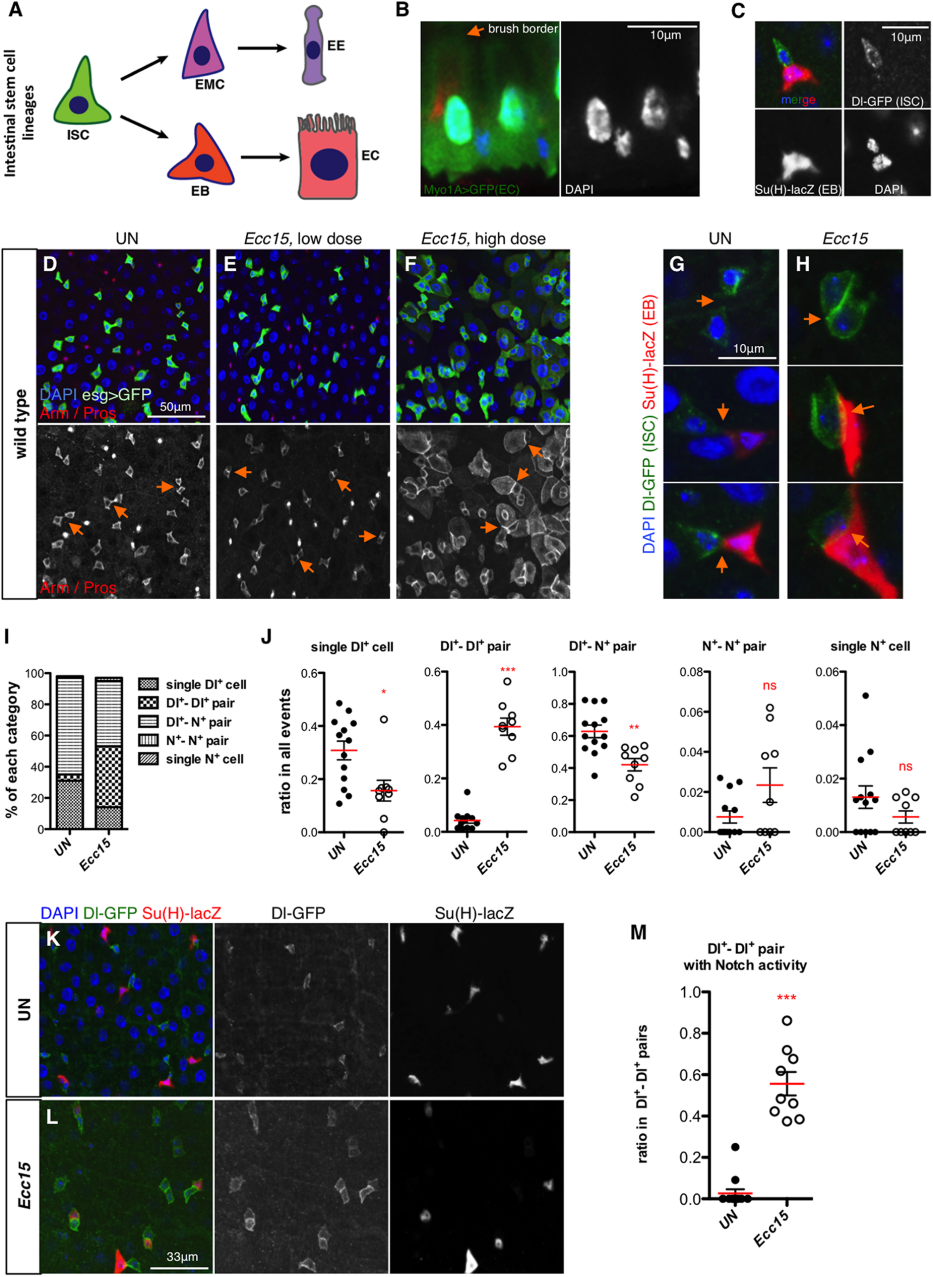
Increased progenitor contact is a general feature of regenerating intestine. (**A**) Current model of intestinal stem cell (ISC) lineages. Cell types in the *Drosophila* midgut include ISC, enterocyte (EC) and its precursor enteroblast (EB), enteroendocrine cell (EE) and its precursor enteroendocrine mother cell (EMC). (**B**) Basal-apical (bottom to top) organization of the *Drosophila* midgut epithelium. ECs are visualized by *Myo1A>GFP* (Green) and brush border is indicated. (**C**) A typical ISC-EB pair in wild type midgut of an unchallenged fly. ISC is detected with *Dl-GFP* (green) and EB with *Su(H)-lacZ* (red). (**D-F**) Representative intestines of unchallenged (D) flies, flies orally infected with a low dose (E) or a high dose of *Ecc15* (F). (**G-H**) Representative progenitor pairs in unchallenged control (G) and *Ecc15*-infected (H) intestines. Junctions between progenitor pairs are indicated with orange arrows in (D-H). (**I-J**) Summary of all the progenitor combinations in unchallenged control (n = 969, from 13 guts) and *Ecc15*-infected (n = 654, from 9 guts) intestines, and ratios of each category (including single *Dl-GFP*^*+*^ cell, *Dl-GFP*^*+*^—*Dl-GFP*^*+*^ pair, *Dl-GFP*^*+*^—*Notch*^*+*^ pair, *Notch*^*+*^*—Notch*^*+*^ pair and single *Notch*^*+*^ cell, from left to right) in all the events under both conditions. (**K-L**) Representative images of unchallenged control (K) and *Ecc15*-infected (L) intestines. Note that the increase in progenitor contact and presence of many *Dl-GFP*^*+*^—*Dl-GFP*^*+*^ pairs with one cell showing weak Notch activity are detected in (L). (**M**) Ratio of *Dl-GFP*^*+*^—*Dl-GFP*^*+*^ pairs with Notch activity (n = 42 in control and 259 in *Ecc15* challenged guts). Each dot represents one gut.

In the adult *Drosophila* midgut, ISCs differentiate into either polyploid absorptive enterocytes (ECs) or diploid secretory enteroendocrine cells (EEs) (Fig 1A). Recent studies indicated that EC and EE are generated through distinct mechanisms [19–21]. A post-mitotic and intermediate differentiating cell called enteroblast (EB) is differentiated into EC in a Notch-dependent manner [22, 23], while the production of EE through a so-far not molecularly characterized enteroendocrine mother cell (EMC) requires only low levels of Notch signaling [24]. ISCs and EBs (referred to as progenitor cells) reside basally next to the visceral muscles, while ECs cover the apical brush border (Fig 1B). In both flies and mammals, Notch signaling plays the same central roles in the choice of an absorptive or secretory fate in the intestinal lineages [25] [26]. *Drosophila* ISCs express the Notch ligand, Delta (Dl), which turns on Notch activity in its sibling cells for EB fate commitment [23, 25] (Fig 1C). Moreover, JAK/STAT signaling [14, 27], the transcription factors Escargot (Esg) [28–30], Sox21a [31, 32], GATAe [33], and Dpp signaling [34–36] have recently been shown to regulate progenitor differentiation. While stem cell proliferation has been the focus of most studies, the cellular mechanisms that mediate proper conversion of the expanded stem cell pool into mature intestinal cells especially during regeneration, are currently missing. Moreover, an integrated view of intestinal regeneration has not been established.

Here, we investigate the cellular and genetic basis underlying efficient differentiation of progenitor cells during intestinal regeneration. Our data uncover that enhanced cell-cell contact between an ISC and its differentiating daughter, consolidated by cell adhesion molecules, is required for efficient Notch signaling and rapid progenitor differentiation into EC during regeneration. We further identify a regulatory cascade involving, sequentially, JAK/STAT signaling, Sox21a and GATAe, that functions in EBs and is required for rapid differentiation. Our integrated study of intestinal regeneration provides new insights into stem cell differentiation that likely apply to other systems.

## Results

### Increased progenitor contact is a general feature of the regenerating intestine

To understand the molecular and cellular mechanisms underlying intestinal regeneration, we analyzed the behaviors of progenitors in the gut of flies orally infected with the gram-negative bacterium *Erwinia carotovora carotovora 15* (*Ecc15*). Oral infection with *Ecc15* causes damage to the intestinal epithelium, which is quickly repaired through activating ISC proliferation and progenitor differentiation to maintain tissue integrity [15]. Unless otherwise noted, we focused our study on the anterior midgut, a region where the relatively low overall cell density allows better identification of individual cells. Interestingly, progenitor pairs with extended contact were observed in intestines following infection (Fig 1D and 1E), visualized by β-catenin staining (Armadillo (Arm) in *Drosophila*). Most differentiating EBs were in extended contact with at least one cell with strong *esg>GFP* signal (Fig 1F). Furthermore, ingestion of dextran sulfate sodium (DSS), which damages the intestinal epithelium and activates regeneration [16], also led to formation of progenitor pairs with extended cell-cell contact (S1A Fig). Since extended progenitor contact was not observed in basal homeostatic conditions, these results indicate that increased progenitor contact area is a general feature of the regenerating intestine.

Both *Ecc15* infection and DSS treatment activate stem cell proliferation. To investigate if division of stem cells is required for the formation of extended progenitor contact, we used colcemid, a microtubule-depolymerizing drug, which blocks dividing cells in metaphase. The presence of colcemid suppressed the formation of extended contact that is normally induced by *Ecc15* infection (S1B and S1C Fig). This suggests that during regeneration stem cells first proliferate before generating progenitors with increased cell-cell contact. However, the formation of extended cell-cell contact was not affected in *Sox21a* mutant gut where EB to EC differentiation is blocked (S1D and S1E Fig). Moreover, clusters of ISCs induced upon expression of a *Notch* RNAi using the progenitor specific driver *esg*^*TS*^ also showed extended contact (S1F Fig). Thus, the formation of increased cell contact during regeneration appears to be a stem cell intrinsic behavior, which occurs independently of developmental signals regulating terminal differentiation.

### Regenerating intestines show an increase in Dl-GFP^+^-Dl-GFP^+^ pairs with extended contact

Dl/Notch signaling plays a central role in determining the ISC and EB cell fate and is further involved in EB differentiation into EC. Since this signal transduction requires cell-cell contact between the signaling sending and receiving cells, the change in contact area likely affects Dl/Notch signaling dynamics [37]. We hypothesized that the extended progenitor contact observed in epithelial damage-induced regenerating intestines could promote efficient differentiation by enhancing Dl/Notch signaling. This led us to further analyze Notch signaling state in progenitor pairs of regenerating intestines by applying cell-type specific markers.

To unambiguously identify ISCs, we used an endogenous *Dl-GFP* fusion line with an ISC restricted expression [38] (Fig 1G). EBs were visualized by *Su(H)-lacZ*, a reporter gene of Notch activity [39, 40]. We first confirmed the increase in progenitor contact upon bacterial infection (Fig 1G and 1H). In line with previous results [5, 25, 41], ISCs in unchallenged conditions largely undergo asymmetric division, which generates another self-renewing ISC (*Dl-GFP*^*+*^) and a committed EB (*Su(H)-lacZ*^*+*^) (Fig 1G and 1I). When we quantified all progenitor combinations, including single *Dl-GFP*^*+*^ cells, *Dl-GFP*^*+*^*—Dl-GFP*^*+*^ pairs, *Dl-GFP*^*+*^*—Notch*^*+*^ pairs, *Notch*^*+*^*—Notch*^*+*^ pairs and single *Notch*^*+*^ cells, in both unchallenged and bacteria-infected (*Ecc15*, 12 hours post infection) intestines, we uncovered a significant increase of the *Dl-GFP*^*+*^*—Dl-GFP*^*+*^ pairs in infected guts (Fig 1I and 1J). Notably, the ratio of *Dl-GFP*^*+*^*—Dl-GFP*^*+*^ pairs increased from 5% in unchallenged intestines to around 40% in infected guts. This change was accompanied by a reduction in the proportion of single *Dl-GFP*^*+*^ cells in regenerating intestines, suggesting that most of them had recently divided. We also observed a drop in the ratio of *Dl-GFP*^*+*^*—Notch*^*+*^ pairs, from 62% to 42%. Collectively, these data are consistent with the notion that increased contact directly arises from newborn progenitor pairs that have just completed mitosis.

**Fig 2:**
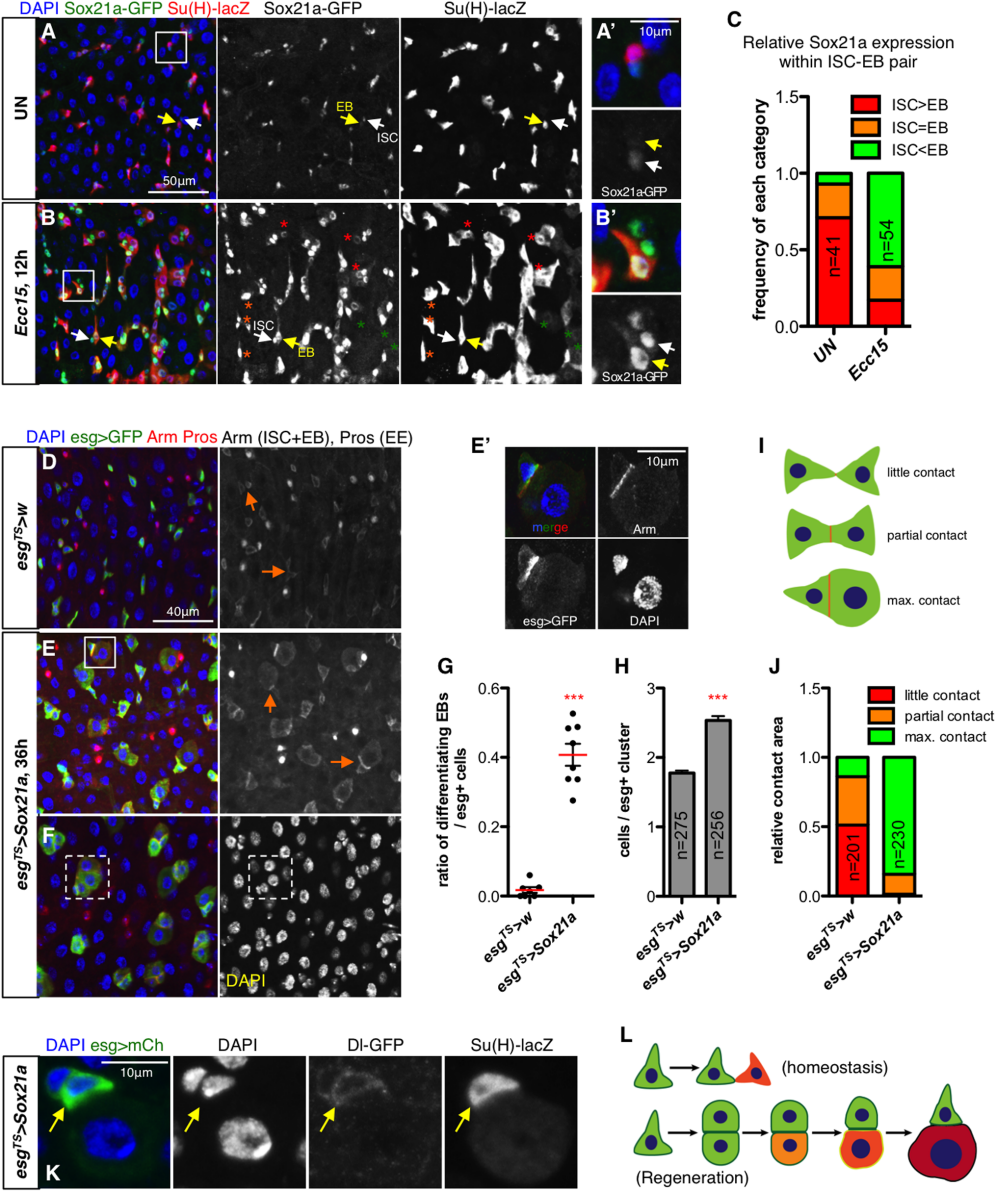
Sox21a regulates intestinal regeneration. (**A-B** and **A’-B’**) Confocal images of unchallenged control (UN, A) and *Ecc15*-infected (B) midguts carrying *Sox21a-GFP* and *Su(H)-lacZ*. Representative ISCs (*Sox21a-GFP*^*+*^ *Su(H)-lacZ*^—^) are labeled with white arrows, and EBs (*Su(H)-lacZ*^*+*^) with yellow arrows. Some EBs are further indicated with stars of different colors in (B): *Sox21a-GFP*^*high*^ *Su(H)-lacZ*^*high*^ (orange), *Sox21a-GFP*^*low*^ *Su(H)-lacZ*^*high*^ (red) and *Sox21a-GFP*^*negative*^ *Su(H)-lacZ*^*low*^ (green). (**C**) Quantification of relative *Sox21a-GFP* intensity within individual ISC-EB pairs from intestines shown in A (n = 41 pairs, from 5 guts) and B (n = 54 pairs, from 7 guts). (**D-F** and **E’**) Representative intestines of wild-type flies (D) or flies over-expressing *Sox21a* in progenitors using *esg*^*TS*^ driver for 36 hours at 29°C (E, E’ and F). Note that Armadillo (Arm) is evenly distributed along the plasma membrane in (D) but is highly enriched at the interface between progenitor cells in (E) (arrows). Prospero (Pros, red, nuclear) marks EEs. Close-up view of a progenitor pair outlined in (E) is shown in (E’). An *esg>GFP*^*+*^ nest containing multiple cells is outlined in (F). (**G**-**H**) Quantification of the ratio of differentiating EBs out of all the *esg>GFP*^*+*^ cells (G) and the average number of cells in each *esg>GFP*^*+*^ nest (H) in intestines of wild-type flies (*esg*^*TS*^*>w*) or flies over-expressing *Sox21a* in progenitors (*esg*^*TS*^*>Sox21a*) for 36 hours. (**I-J**) Schematic progenitor contact categories (I) and the distribution of each contact category in intestines of wild-type flies (*esg*^*TS*^*>w*) or flies over-expressing *Sox21a* in progenitors (*esg*^*TS*^*>Sox21a*) for 36 hours (J). Nuclei are stained by DAPI. (**K**) Representative image of progenitors from *esg*^*TS*^*>Sox21a* flies carrying both ISC and EB markers after 36 hours of transgene expression. Progenitors are shown with *esg>mCherry* (green). Yellow arrow indicates the *Dl-GFP*^*+*^ cell with Notch activity. (**L**) Schematic representation of modes of ISC division and cell fate commitment during homeostasis and regeneration. The presence of strong junction between the two progenitors promotes Notch signaling allowing rapid differentiation. Green lines indicate Dl, and red fillings denote Notch activation. Each dot represents one gut.

Interestingly, 57% of the *Dl-GFP*^*+*^*—Dl-GFP*^*+*^ pairs had one cell showing weak but specific Notch activity in *Ecc15*-infected intestines as revealed by the expression of the *Su(H)-lacZ* reporter (Fig 1K–1M; S2A and S2B Fig). Use of an antibody against Dl confirmed that both cells, including the one with weak *Su(H)-lacZ* expression, were indeed stem cells as defined by the expression of the Dl marker (S2C Fig). We further excluded the possibility of EE differentiation, since the EE marker Prospero (Pros) was never observed in such *Dl-GFP*^*+*^*—Dl-GFP*^*+*^ pairs with Notch activity (n>50) (S2D Fig). Importantly, ISCs undergoing mitosis were never found to express *Su(H)-lacZ* reporter (n>30) (S2E Fig), indicating that Notch activity was established in one cell of a newly formed *Dl-GFP*^*+*^*—Dl-GFP*^*+*^ pair after mitosis (S2F and S2G Fig). Although *Dl-GFP*^*+*^*—Dl-GFP*^*+*^ pairs formed during infection can arise either from single *Dl-GFP*^+^ cells or from *Dl-GFP*^*+*^*—Notch*^*+*^ progenitor pairs, these results support a symmetric expansion of the Dl^+^ cell pool followed by diverting a subset of them to be committed into EB and quickly differentiated, likely due to the presence of the extended cell-cell contact (Fig 2L).

### *Sox21a* is induced in intestinal progenitors during regeneration

We and others have previously shown that the transcription factor Sox21a is both necessary and sufficient for the differentiation of EB to EC [31, 32]. This was supported by the observations that ISC progenies are blocked at the EB stage in the absence of *Sox21a*, while overexpressing *Sox21a* in progenitors induced their precocious differentiation into mature EC. However, it is not yet established through which mechanisms Sox21a regulates progenitor differentiation, especially during regeneration following intestinal damage.

To further analyze the role of *Sox21a* in EB differentiation, we first monitored its expression levels in intestinal cells at different stages of their differentiation using a sGFP (superfolder green fluorescent protein)-tagged *Sox21a* transgene that is controlled by its own regulatory sequences [42]. Since it has been shown that *Sox21a* is expressed in both ISC and EB, cells that express *Sox21a-GFP* but not *Su(H)-lacZ* reporter are expected to be ISCs. As expected for a transcription factor, *Sox21a-GFP* signal was localized to the nuclei (Fig 2A and 2B). In unchallenged 5–7 day-old adults, *Sox21a-GFP* was expressed in both ISC and EB, with higher levels in ISC than in EB within an ISC-EB pair (Fig 2A, 2A’ and 2C). Oral infection with *Ecc15* induced a marked increase of *Sox21a-GFP* expression in the progenitors (Fig 2B and 2B’). However, quantification of the relative intensity of *Sox21a-GFP* levels between cells within each ISC-EB pair indicated that regenerating intestines now expressed stronger *Sox21a-GFP* in the EB than in its sibling ISC (Fig 2C). Careful examination further revealed that *Sox21a-GFP* levels started declining in middle-sized EBs at a stage when Notch activity remained high. Moreover, *Sox21a-GFP* expression was totally shut down before the Notch activity reporter (Fig 2B).

Intestinal regeneration can be triggered by activating JNK or Ras/MAPK signaling in progenitors [12, 43, 44]. Interestingly, activating either pathway in progenitor cells with *esg-Gal4 tub-Gal80*^*ts*^ for 36 hours also elevated *Sox21a-GFP* expression (S3A–S3E Fig), consistent with a previous observation [45]. However, akin to infection, stronger *Sox21a-GFP* expression was again seen in EBs, supporting the specific role of Sox21a in EB for EC differentiation. Collectively, this analysis shows that *Sox21a* displays a dynamic expression pattern, which coincides with the process of intestinal progenitor differentiation.

### Expressing *Sox21a* efficiently promotes progenitor differentiation

As shown previously [31], expressing *Sox21a* in progenitor cells with the *esg*^*TS*^ driver for only 36 hours led to their differentiation into EC (Fig 2D–2F). Thus, over-expressing *Sox21a* provides a useful framework to unravel the sequence of events underlying stem cell differentiation into mature ECs. Taking advantage of this approach, we monitored the cellular changes resulting from overexpressing *Sox21a* in progenitors. Cells that were precociously differentiating towards ECs (termed “differentiating EBs”) showed an increase in cell size, became polyploid and expressed Pdm1, a marker for ECs [31]. Differentiating EBs accounted for around 40% of the *esg>GFP*^+^ progenitors expressing *Sox21a*, while they were barely seen in midgut progenitors of wild type flies (Fig 2G). They retained a weak *esg>GFP* signal and were located at a similar basal position as genuine ISC/EB (S3F Fig). In addition, each *esg>GFP*^*+*^ nest contained a slightly increased number of cells (Fig 2F and 2H), consistent with the notion that *Sox21a* also promotes a low level of ISC proliferation.

In homeostatic guts, most progenitor cells have limited membrane contact (Fig 2D). In *Sox21a* overexpressing guts, nearly all the differentiating EBs displayed an extended plasma membrane contact, with at least one cell with a strong *esg>GFP* signal (Fig 2E and 2E’; S3F Fig). Increased membrane contact was also reflected by the increased GFP signal at the membrane in progenitor cells expressing the membrane-tethered mCD8::GFP (Fig 2E’). To quantify changes in membrane contact, we measured contact area relative to the size of the smaller cell in more than 200 progenitor pairs. Maximal cell-cell contact between progenitors was reached as early as 36 hours after *Sox21a* expression was induced (Fig 2I and 2J). Similarly to the situation observed with infection, expressing *Sox21a* using the progenitor driver *esg-Gal4 tub-Gal80*^*ts*^ also induced *Dl-GFP*^*+*^*—Dl-GFP*^*+*^ pairs with one cell exhibiting Notch activity with a high frequency (Fig 2K).

### Sox21a promotes progenitor differentiation by enhancing Dl/Notch signaling

**Fig 3:**
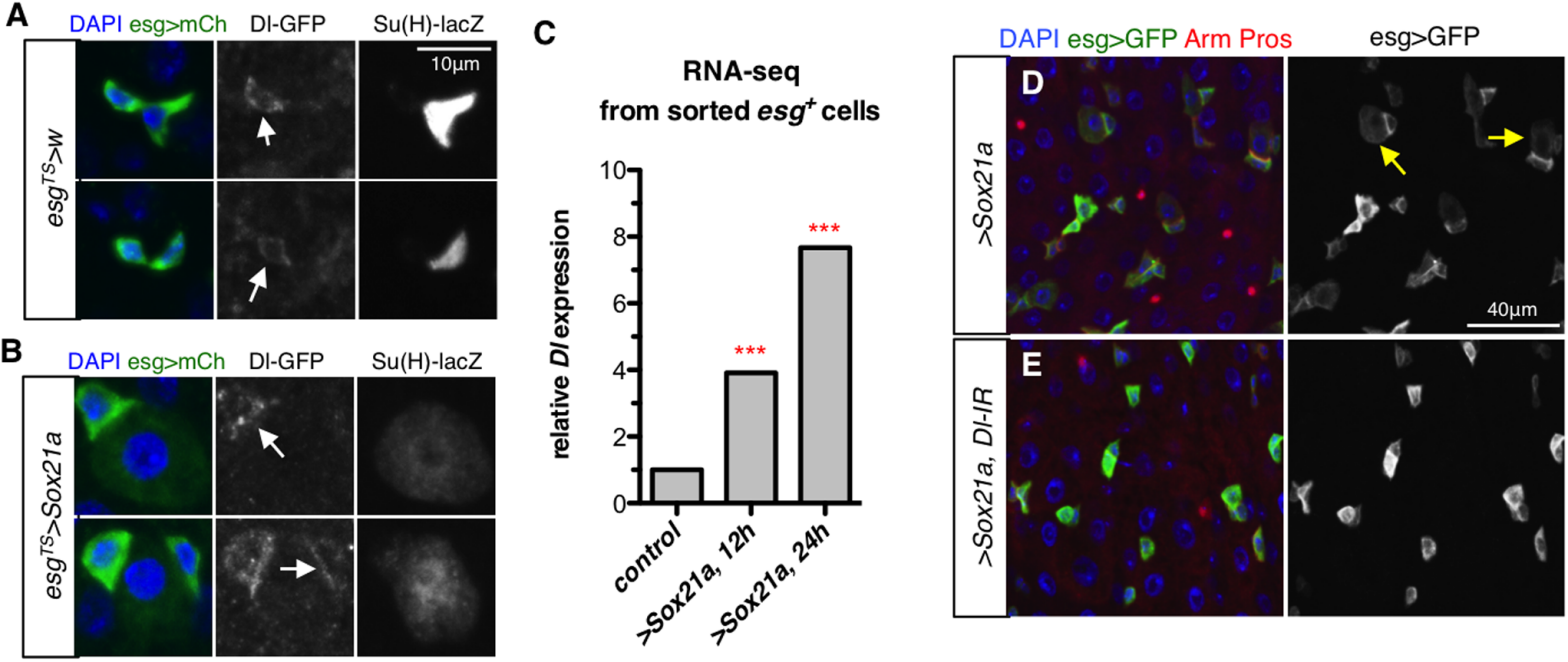
Sox21a promotes differentiation by enhancing the Dl/Notch signaling. (**A-B**) ISC-EB pairs in control (*esg*^*TS*^*>w*) and *Sox21a*-expressing (*esg*^*TS*^*>Sox21a*) flies. ISCs are detected with *Dl-GFP*, EBs with *Su(H)-lacZ* and progenitors with *esg>mCherry* (green). Arrows indicate ISCs in (A) and highlight the ISC-EB contact in (B). (**C**) Relative *Dl* mRNA levels in control and *Sox21a*-expressing progenitors from an RNA-seq experiment using sorted *esg>GFP*^*+*^ cells (n = 50 midguts/group, in two biological replicates, Robinson and Smyth Exact test, ***p < 0.001). *esg*^*TS*^*>Sox21a* flies were shifted to 29°C for 12 or 24 hours to induce Sox21a expression. (**D-E**) Confocal images of intestines expressing *Sox21a* alone (D) or simultaneously with a *Dl-RNAi* construct (E) using *esg*^*TS*^ for 36 hours. Arrows in (D) indicate some differentiating EBs, which are not present upon depleting *Dl* (E).

**Fig 4:**
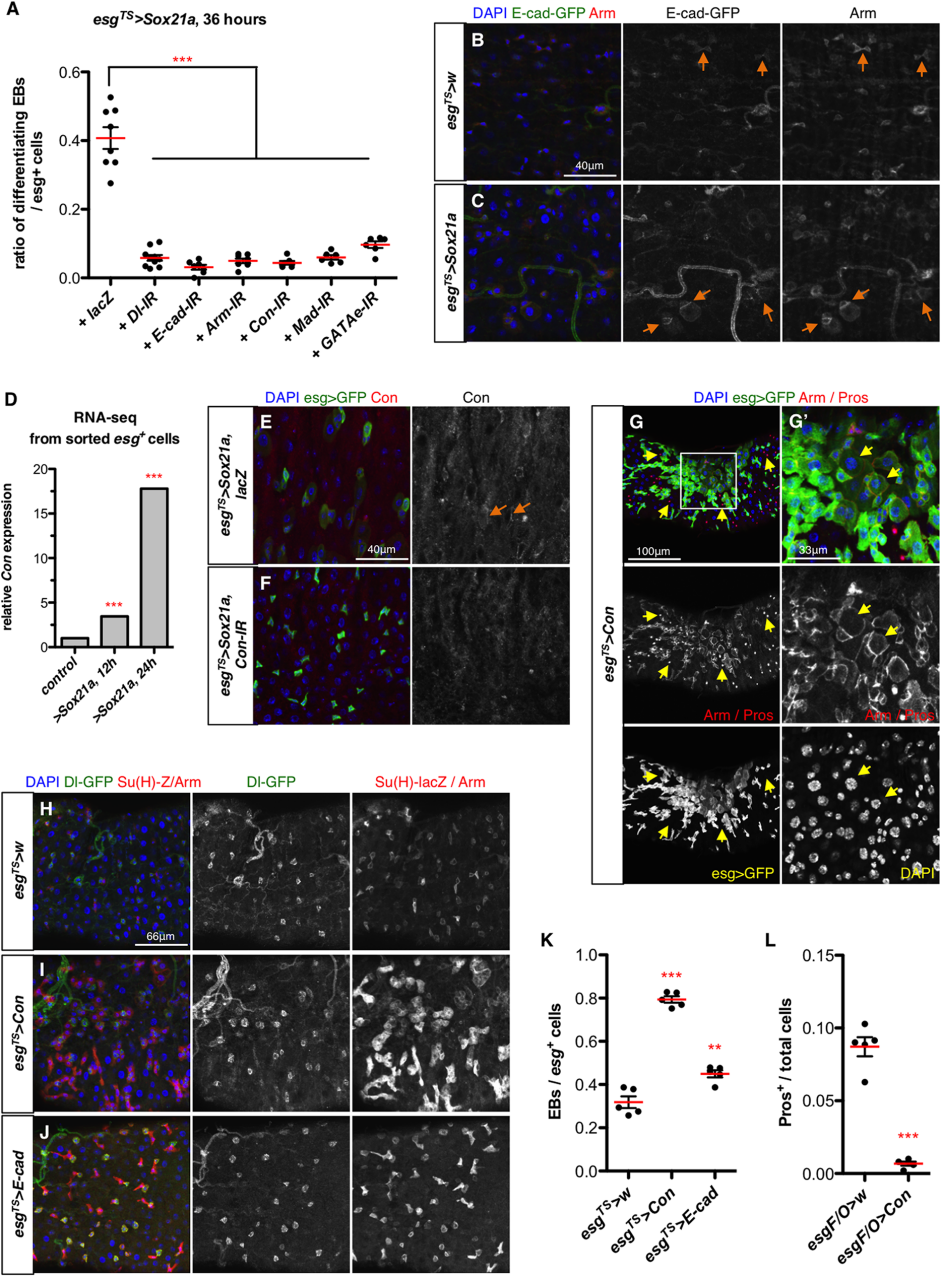
Cell adhesion molecules are required for *Sox21a*-induced differentiation. **(A)** Quantification of the ratio of differentiating EBs out of all the *esg>GFP*^*+*^ cells in intestines of flies over-expressing *Sox21a* in progenitors (*esg*^*TS*^*>Sox21a*) with co-depletion of the indicated genes with RNAi for 36 hours. (**B-C**) Localization of E-cad (revealed with an endogenous *E-cad-GFP* fusion) in both control (B) and *esg*^*TS*^*>Sox21a* intestine (C). E-cad/Arm junctions are indicated by orange arrows. (**D**) Relative *Con* mRNA levels in control and *Sox21a*-expressing progenitors from the RNA-seq experiment described in Fig 3C. (**E-F**) Connectin (Con) is enriched in the progenitor contact in *esg*^*TS*^*>Sox21a* intestines (E, orange arrows) by applying an antibody against Connectin, but is not detected in *esg*^*TS*^*>Sox21a* intestines co-depleted for *Connectin* by RNAi (F). Progenitors (*esg>GFP*) and nuclei (DAPI) are shown. (**G-G’**) Progenitor-specific expression of *Connectin* using *esg*^*TS*^ for 4 days. The region outlined in (G) is shown in (G’) with higher magnification. Note the absence of EEs (Pros^+^) from the region with big clusters of *esg>GFP*^+^ cells, some of which are differentiating towards EC. Some differentiating EBs are indicated with yellow arrows (G’). **(H-K)** representative images of midgut of control flies (H) or flies overexpressing *Connectin* (I) or *E-cad* (J) with *esg*^*TS*^ for 4 days. ISCs express Dl-GFP (in green); EBs are marked with *Su(H)-lacZ* reporter (cytoplasmic, in red). Quantification of the ratio of EBs out of all the progenitors (ISC+EB) is shown in (K). **(L)** Quantification of the ratio of EEs (Pros^+^) in all the midgut cells in defined areas of the anterior midgut of flies with indicated genotype and shifted to 29°C to induce transgene expression for 7 days. Each dot represents one gut.

The observation that over-expressing *Sox21a* induces progenitors to differentiate towards ECs rather than EEs [31], suggests that Sox21a could enhance Notch activity. To test this idea, we analyzed how Sox21a impacts Notch signaling. Interestingly, *Dl-GFP* signal was enriched at the extended ISC-EB contact area upon *Sox21a* expression, in contrast to their even distribution in wild-type ISCs (Fig 3A and 3B). Furthermore, transcriptomic analysis using FACS-sorted progenitors revealed a four to seven fold increase of *Dl* mRNA in intestinal progenitors expressing *Sox21a* for only 12 or 24 hours (Fig 3C; S1 Table). The increase of *Dl* mRNA levels was further validated by quantitative PCR (qPCR) from dissected guts (S4 Fig). Thus, expressing *Sox21a* in progenitors increased *Dl* transcription and the amount of Dl protein at the ISC-EB interface, which are both expected to reinforce efficient Notch signal transduction. Finally, knocking down *Dl* with two independent RNAi lines both suppressed *Sox21a*-induced differentiation (Figs 3D and 3E and 4A; S5A and S5B Fig). Collectively, our data indicate that Sox21a mediates rapid differentiation at least in part by up-regulating *Dl* and by increasing progenitor contact, two features likely to enhance Notch signaling.

### Cell-cell adhesion is essential for *Sox21a*-induced differentiation

Extended progenitor contact in regenerating guts suggested that cell adhesion molecules might be involved in rapid progenitor differentiation. E-cadherin (E-cad) forms a complex with β-catenin at adherens junctions and mediates homophilic cell-cell adhesion [46]. Using an endogenous *E-cad-GFP* fusion, we first confirmed that E-cad was enriched at the ISC-EB interface in *Sox21a* expressing intestine. Similarly, the localization of Arm was almost identical to E-Cad (Fig 4B and 4C). Simultaneous depletion of either *E-cad* or *arm* for 36 hours completely suppressed the precocious differentiation phenotype seen in intestines expressing *Sox21a* (*esg*^*TS*^*>Sox21a*) (Fig 4A; S5C and S5D Fig). In these intestines, progenitor pairs displayed reduced contact confirming an essential role of E-cad in the formation of extended cell-cell contact during rapid differentiation. Since E-cad can impact Wg/Wnt signaling [47], the phenotype observed was possibly due to a requirement of Wg signaling for *Sox21a*-induced differentiation. However, neither activating (with constitutively active β-catenin) nor blocking Wg signaling (with dominant-negative Pangolin) affected *Sox21a*-induced differentiation (S5H and S5I Fig). We conclude that E-cad-mediated cell-cell adhesion is required for *Sox21a*-induced differentiation, independently of Wg signaling, in line with a previous report [48].

Furthermore, another cell adhesion molecule, Connectin (Con), which mediates homophilic cell-cell adhesion both *in vitro* and *in vivo* [49, 50], was up-regulated in progenitor cells expressing *Sox21a* in our progenitor-specific transcriptomic analysis (Fig 4D; S1 Table). Using an antibody against Connectin, we confirmed its enrichment at the extended contact between progenitors in *esg*^*TS*^*>Sox21a* gut (Fig 4E; S6A Fig). Like E-cad, Connectin was also crucial for *Sox21a*-induced differentiation, as its depletion abolished the occurrence of differentiating EBs (Fig 4A and 4F; S5E Fig).

To determine to which extent cell-cell adhesion can contribute to differentiation, we also overexpressed *Connectin* in progenitors. As expected, overexpressing *Connectin* using *esg*^*TS*^ altered the morphology of progenitor cells with the formation of interconnected progenitors, sometimes forming big clusters (Fig 4G; S6A–S6C Fig). These changes were also associated with an increase in ISC proliferation but not a blockage of EB differentiation (Fig 4G’; S6H Fig). Consequently, progenitor tumors were not observed in *esg*^*TS*^*>Connectin* intestines despite the presence of large *esg>GFP*^*+*^ clusters. Surprisingly, many progenitors in *esg*^*TS*^*>Connectin* intestines displayed the same characteristics of differentiating EBs of *esg*^*TS*^*>Sox21a* intestines, including a weak *esg>GFP* signal, increased cell size, and extended contact with neighboring *esg>GFP*^strong^ progenitors (Fig 4G’). Moreover, regions with clusters of *esg>GFP*^*+*^ cells were devoid of EEs and progenitors in such regions were differentiating towards ECs as judged from their large cell size and polyploid nuclei (Fig 4G and 4G’; S6B and S6C Fig). Further experiments indicated that *Connectin* overexpression in the progenitors increased the expression levels of Notch activity reporter *Su(H)-lacZ* (Fig 4I) and promoted EB-EC differentiation rather than EE differentiation (Fig 4H–4L; S6I and S6J Fig). Therefore, an increase in cell adhesion by over-expressing *Connectin* in progenitors can promote their differentiation toward ECs, by enhancing Notch activity. Nevertheless, results obtained with the *esgF/O* system [14] did not support an essential role of E-cad or Connectin in basal intestinal turnover (S6K–S6M Fig). Unexpectedly, knocking-down *Connectin* in the progenitors induced both mild stem cell proliferation and progenitor differentiation in the absence of a challenge (S6D–S6H Fig), suggesting Connectin may have other functions in the maintenance of ISC under normal conditions. We conclude that the formation of extended cell contact between progenitors through adhesion molecules is required for Sox21a-induced rapid differentiation. Importantly, we show that this process is specifically required for the rapid differentiation of progenitors but not for basal low-level intestinal turnover during homeostatic conditions. Thus, our study reveals specific mechanisms that have evolved to accelerate the differentiation program.

### Sox21a acts in parallel with Notch signaling for terminal differentiation

Having analyzed the cellular changes that enhance Notch signaling activity in the ISC-EB transition during intestinal regeneration, we went on to investigate how Sox21a contributes to the processes of differentiation from EB to EC. In *Drosophila*, *Notch* deficient stem cells over-proliferate, leading to the formation of tumors composed mostly of ISCs and intermingled with EEs [22, 23, 25, 51], while the over-activation of Notch signaling drives progenitors to differentiate into ECs [22, 25]. The similarities between the function of Notch signaling and that of Sox21a in terminal differentiation led us to investigate their relationship.

**Fig 5:**
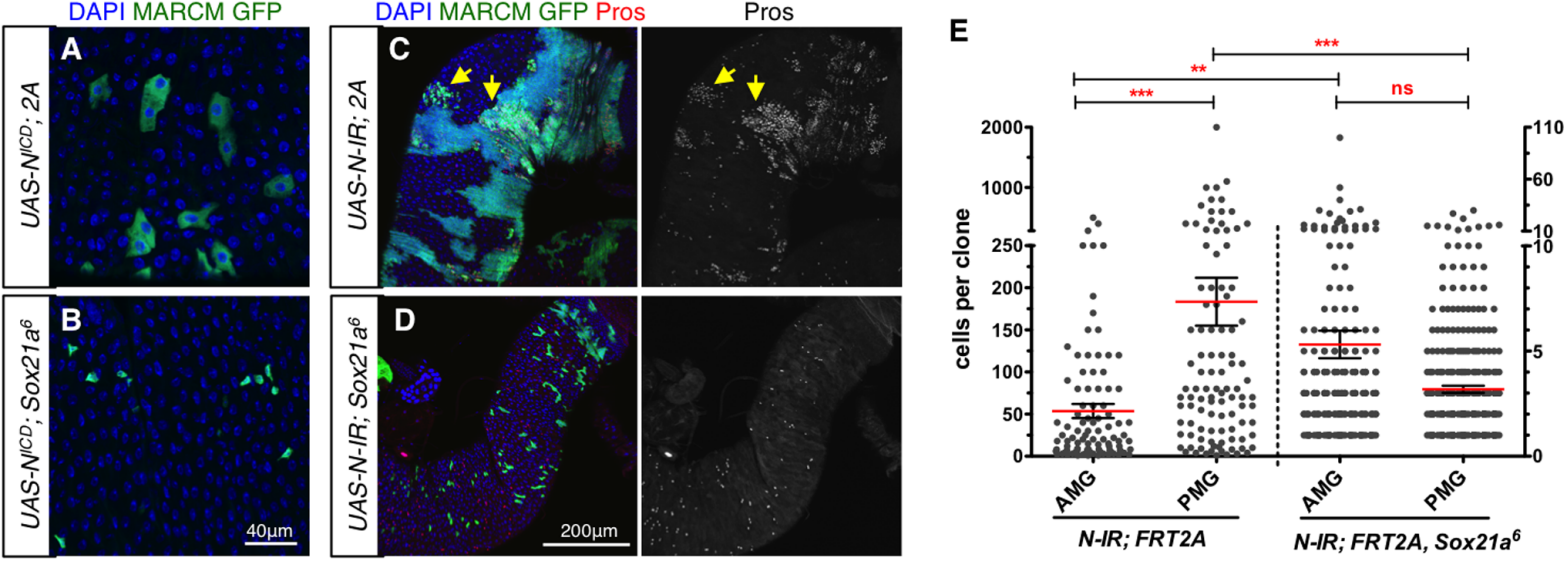
Sox21a acts in parallel with Notch signaling for terminal differentiation. (**A-D**) Control (A and C) or *Sox21a* mutant (B and D) MARCM clones (positively labeled by GFP) in the posterior midgut (PMG) overexpressing *UAS-Notch*^*ICD*^ to activate Notch signaling (A and B) or over-expressing a *UAS-Notch-IR* construct to deplete Notch (C and D), are analyzed 7 days after clone induction (ACI). ECs are identified by their large cell size (A), and EEs express Prospero (yellow arrows in C). Different UAS-linked transgenes are combined either to a *FRT2A* chromosome to generate control clones (A and C) or to *FRT2A*, *Sox21a*^*6*^ chromosome to make *Sox21a* mutant clones (B and D). (**E**) Quantification of the size of *Notch* deficient clones (using a Notch RNAi construct) in wild type or *Sox21a*^*6*^ flies. Clone size was monitored in the anterior midgut (AMG) and the posterior midgut 12 days ACI at 18°C. Numbers of clones scored are 100, 104, 207 and 373, respectively from left to right, derived from 10 guts. Cell counts for *Sox21a* mutant clones should apply the *y-axis* on the right side of the figure. Note that a stronger inhibition of the *Notch* tumor growth by loss of *Sox21a* was observed in the PMG than in the AMG, agreeing with a previous finding that *Sox21a* is essential for ISC proliferation only in the PMG, but not in the AMG.

While *Sox21a* is expressed in both ISC and EB, Notch activity is only found in EB [22, 23]. Thus, *Sox21a* expression in ISC is independent of Notch signaling. In addition, Notch activity was not blocked in a *Sox21a* mutant [31]. Several observations indicate that Sox21a and Notch signaling function interdependently for terminal differentiation. We first observed that *Sox21a* was required for the differentiation of progenitors into ECs upon Notch over-activation (Fig 5A and 5B), and for the formation of tumors composed of ISCs and EEs in *Notch* deficient clones (Fig 5C–5E). Conversely, the forced differentiation of progenitors into ECs upon expression of *Sox21a* was blocked in the absence of functional Notch signaling (S7A–S7D Fig). Thus, over-expression of *Sox21a* cannot overcome the requirement of Notch signaling for the differentiation of EB. Collectively, this led us to conclude that Sox21a and Notch signaling encompass two parallel systems that need to cooperate to ensure terminal differentiation.

### Sox21a functions downstream of the JAK/STAT pathway in EB differentiation

**Fig 6:**
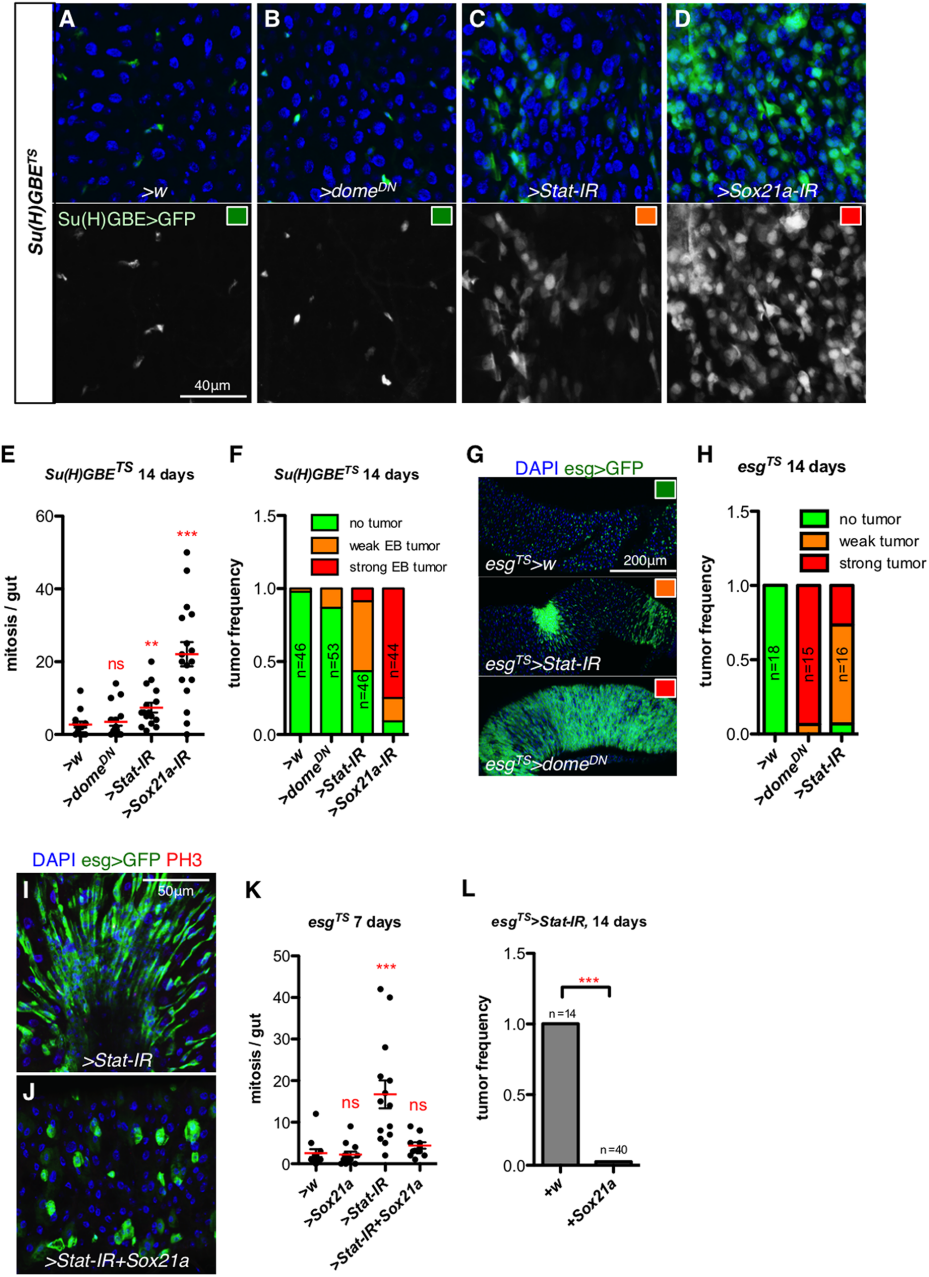
Sox21a functions downstream of JAK-STAT pathway for EB differentiation. (**A-D**) Representative intestines of flies with the indicated genotype observed 14 days after transgene expression using EB-specific driver *Su(H)GBE*^*TS*^. *Su(H)GBE>GFP* (green) labels EBs. (**E**) Quantification of mitotic index in the midgut of flies after knocking down respective factors using EB-specific driver *Su(H)GBE*^*TS*^ for 14 days. (**F**) Quantification of tumor formation capacity in the midgut of flies shown in (A-D). The tumor-grading system is based on the degree of EB accumulation and examples are color-coded and denoted in (A-D). (**G-H**) Representative images of intestines with indicated genotype observed 14 days after transgene expression using the progenitor-specific driver *esg*^*TS*^ (G) and quantification of progenitor tumor frequency (H). The tumor-grading standard is shown in (G). *esg>GFP* (green) labels ISC/EB. (**I-J**) Representative intestines of flies with the indicated genotype observed 7 days after transfer to 29°C allowing transgene expression using *esg*^*TS*^. (**K**) Quantification of mitotic index of midgut from flies with the indicated genotype. (**L**) Quantification of the frequency of intestinal progenitor tumors in *esg*^*TS*^*>Stat-IR* flies and *esg*^*TS*^*>Stat-IR* flies co-expressing *Sox21a* to induce differentiation for 14 days. Each dot represents one gut.

Both Sox21a and JAK/STAT have been shown to be mandatory for the EB-EC differentiation, and knockdown of either of these two factors results in the formation of EB containing tumors. Mechanistically, the formation of these tumors is caused by a feed-back amplification loop whereby the differentiation-defective EBs secrete growth factors stimulating ISC proliferation [14, 27, 31, 32]. We have previously shown that over-expression of *Sox21a* can partially rescue the differentiation defect caused by the loss of JAK/STAT in MARCM clones, suggesting that Sox21a functions downstream of JAK/STAT signaling in EB differentiation [31]. We further analyzed the relationship between JAK/STAT signaling and Sox21a by using this time an EB specific driver, *Su(H)GBE*^*TS*^ (Fig 6A–6D). Knocking down *Sox21a* specifically in EB caused the accumulation of EBs and an increase in ISC mitosis resulting in strong tumor formation, recapitulating the *Sox21a* mutant phenotype (Fig 6D–6F). In contrast, EB-specific depletion of *Stat92E*, the gene encoding the JAK/STAT transcription factor, had only mild consequences with the formation of small-sized EB tumors (Fig 6C, 6E and 6F). Unexpectedly, expressing a dominant-negative form of *Domeless* (*Dome*^*DN*^), the receptor of the JAK/STAT signaling cascade, using the same EB driver did not affect the differentiation process and did not lead to any tumor formation (Fig 6B, 6E and 6F). However, silencing the JAK/STAT pathway in both ISC and EB with *esg*^*TS*^, using the same *UAS-Dome*^*DN*^ or the *UAS-Stat-RNAi* constructs led to the formation of massive progenitor tumors (Fig 6G and 6H). The observations that EB tumor formation requires the inactivation of JAK/STAT in both ISC and EB, and that knock-down of *Sox21a* only in EBs is sufficient to cause tumors, are consistent with the notion that JAK/STAT signaling is required earlier than Sox21a in the course of EB-EC differentiation. Supporting this hypothesis, we observed that expressing *Sox21a* could suppress tumor formation caused by loss of JAK/STAT signaling in progenitors (Fig 6I–6L), indicating that Sox21a can promote progenitor differentiation in the absence of JAK/STAT signaling. Thus, the observations that JAK/STAT signaling functions earlier than Sox21a, and that Sox21a can override the differentiation blockage caused by the loss of JAK/STAT signaling confirm and extend our previous observation based on mosaic analysis [31] that Sox21a acts downstream of JAK/STAT pathway in EB-EC differentiation.

### The Dpp signaling pathway is required in EBs for *Sox21a*-induced differentiation

**Fig 7:**
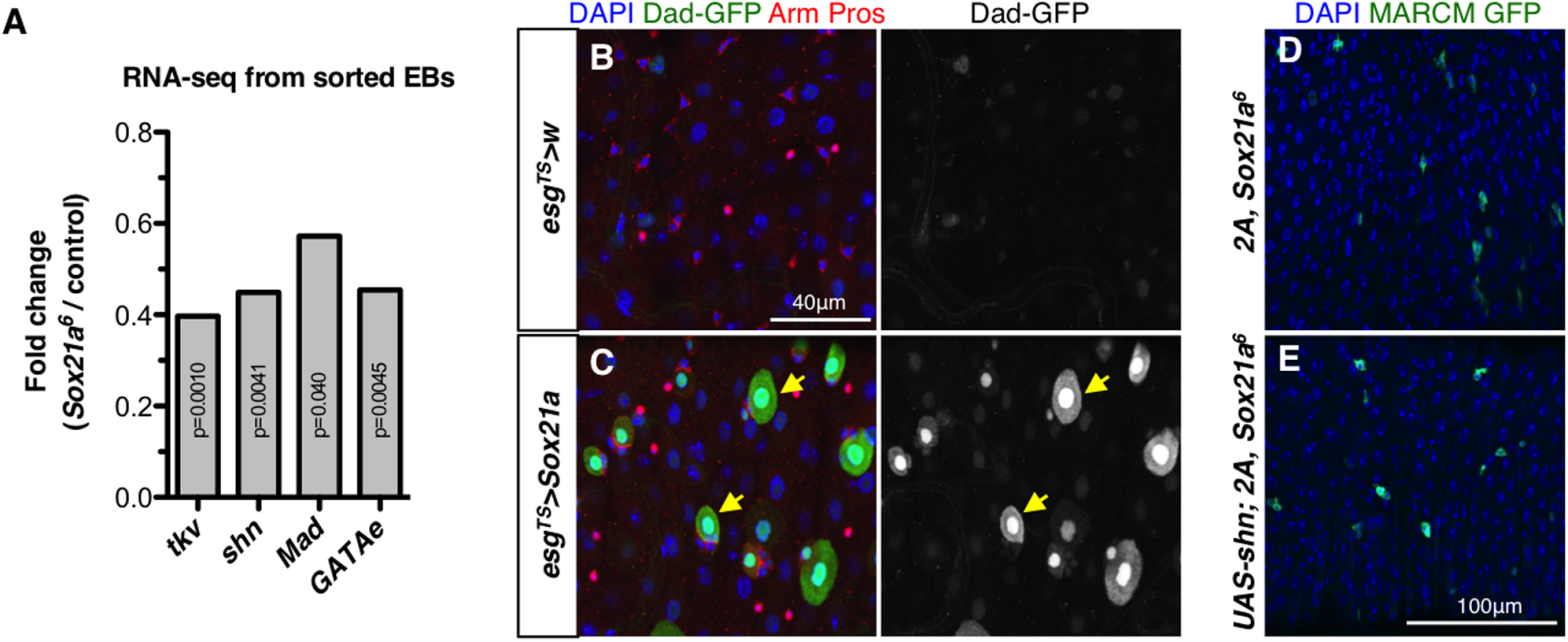
The Dpp signaling is required in EBs for *Sox21a*-induced differentiation. (**A**) Fold changes of respective transcripts (*thickvein* (*tkv*), *schnurri* (*shn*), *Mothers against dpp* (*Mad*) and *GATAe*) in *Sox21a* mutant EBs compared to wild type EBs. (**B-C**) The level of Dpp signaling activity was detected with the *Dad-GFP*^*nls*^ reporter (in green) in wild-type flies (B) or flies over-expressing *Sox21a* in progenitors for 36 hours (C). Some differentiating EBs are indicated with yellow arrows (C). Arm (in red, membrane), Pros (in red, nuclei) and DAPI staining are shown. (**D-E**) *Sox21a* mutant clones (positively labeled by GFP, D) and *Sox21a* mutant clones expressing the Dpp pathway transcription factor Schnurri (E) are analyzed 7 days after clone induction. Note that the blockage in differentiation of *Sox21a* mutant clone cells was not rescued by the *shn* expression.

The role of the Dpp signaling pathway in EB differentiation has been controversial, with studies supporting that it is essential to this process while others suggest it is dispensable [34–36]. The observation that several genes encoding components of Dpp signaling, including the receptor *thickveins* (*tkv*), the transcription factors *schnurri* and *Mothers against dpp* (*Mad*), are down-regulated in *Sox21a* mutant EBs, supported a role of Dpp signaling in EB to EC transition (Fig 7A). We therefore explored its role in the differentiation process and its relationship with Sox21a. Interestingly, Dpp signaling was specifically induced in differentiating EBs in *esg*^*TS*^*>Sox21a* intestines as revealed by a reporter gene of Dpp signaling, *Dad-GFP*^*nls*^ (Fig 7B and 7C). The expression of the *Dad-GFP*^*nls*^ reporter was much stronger in differentiating EBs than in mature ECs that were already differentiated prior to the activation of *Sox21a* (Fig 7C), highlighting a role of Dpp signaling in the EB to EC transition. Importantly, Dpp signaling was mandatory for *Sox21a*-induced rapid differentiation, as depleting the key component *Mad* abolished progenitor differentiation of *esg*^*TS*^*>Sox21a* intestines (Fig 4A; S5F Fig). These results support a role of the Dpp pathway in EB differentiation downstream of Sox21a. Nevertheless, overexpressing the Dpp transcription factor Schnurri, which is known to promote progenitor differentiation into EC in the midgut [36], did not rescue the differentiation defect of *Sox21a* mutant clones (Fig 7D and 7E). We conclude that Dpp signaling is required downstream of Sox21a for rapid differentiation but is not sufficient to rescue *Sox21a* deficiency.

### GATAe is required in EBs for *Sox21a*-induced differentiation

*GATAe* encodes a transcription factor, which is expressed in all the cell types of the fly midgut. It has recently been shown to be important for ISC proliferation and, to a lesser extent, for EB differentiation [33]. It also has a role in ECs to maintain the regionalization of the intestine [33, 52]. Our RNA-seq experiments with FACS-sorted EBs revealed that the expression of *GATAe* was decreased in the absence of *Sox21a* (Fig 7A), suggesting that this transcription factor could contribute to the differentiation program downstream of Sox21a. We therefore investigated in further detail the role of GATAe in the differentiation process and its relationship with Sox21a.

**Fig 8:**
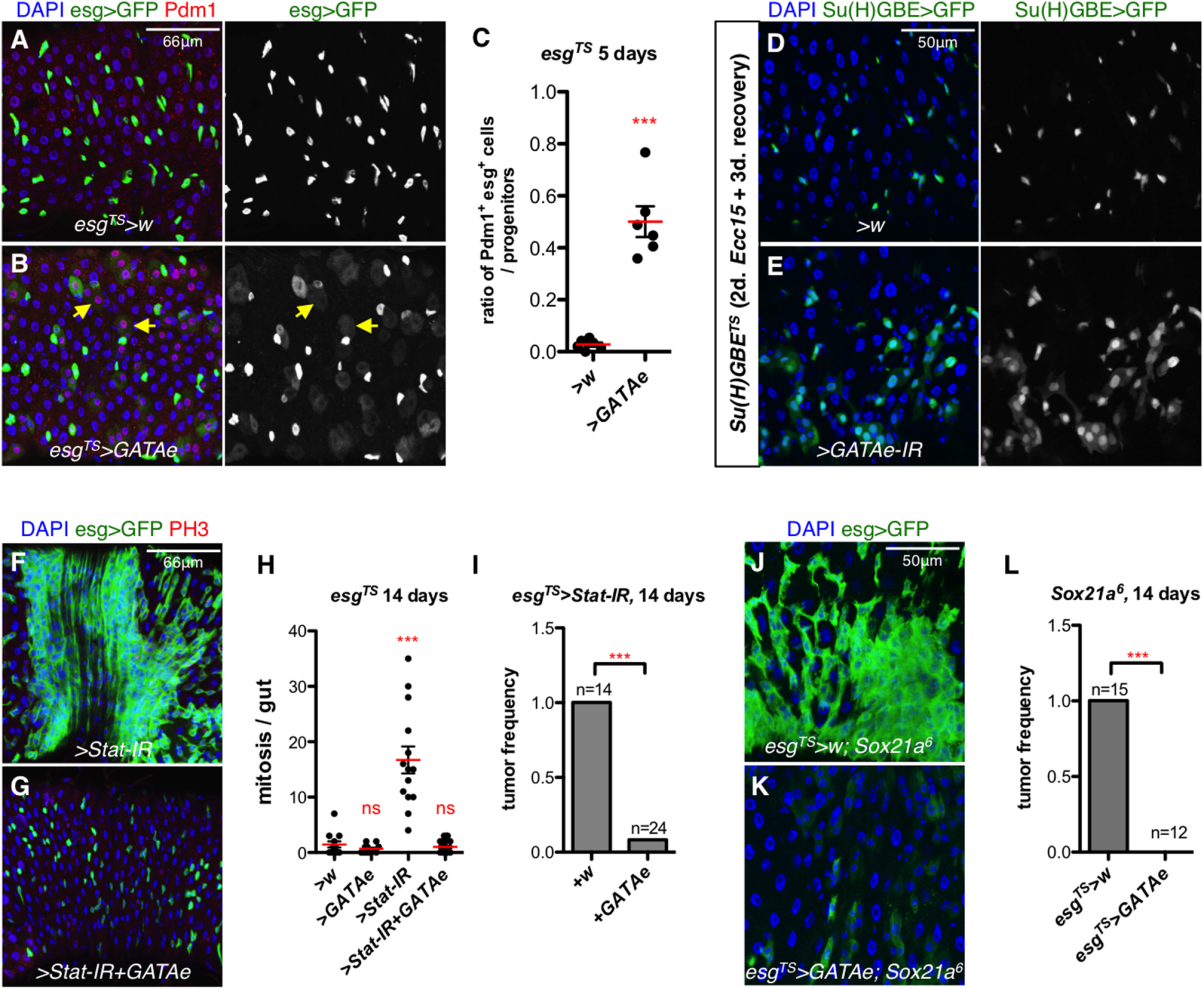
GATAe is required in EBs for *Sox21a*-induced differentiation. (**A-B**) Representative intestines of wild-type flies (A) or flies over-expressing *GATAe* (B) in progenitors using *esg*^*TS*^ driver for 5 days. EC marker Pdm1 is shown in red. Note many differentiating progenitors already express Pdm1 (indicated by arrows). (**C**) The percentage of of *Pdm1*^*+*^ *esg>GFP*^*+*^ cells over all the *esg>GFP*^*+*^ progenitors in both control (*esg*^*TS*^*>w*) and *GATAe* overexpressing intestines (*esg*^*TS*^*>GATAe*). (**D-E**) Intestines of *Su(H)GBE*^*TS*^*>w* (control, D) and *Su(H)GBE*^*TS*^*>GATAe-IR* (E) flies which were shifted to 29°C to induce transgene expression for 3 days, then orally infected with *Ecc15* for 2 days and recovered for 3 additional days. *Su(H)GBE>GFP* (green) labels EBs. (**F-G**) Representative intestines of flies with the indicated genotype observed 14 days after transfer to 29°C allowing transgene expression using *esg*^*TS*^. (**H**) Quantification of mitotic index of midgut from flies with the indicated genotype. (**I**) Quantification of the frequency of intestinal progenitor tumors in *esg*^*TS*^*>Stat-IR* flies and *esg*^*TS*^*>Stat-IR* flies co-expressing *GATAe* for 14 days. (**J-K**) Intestines of *Sox21a* mutant fly and *Sox21a* mutant fly carrying *esg*^*TS*^*>GATAe* transgenes, kept at 22°C for 20 days to allow the formation of progenitor tumors and then shifted to 29°C for 2 days to induce transgene expression. (**L**) Tumor frequency in intestines of *Sox21a* mutant and *Sox21a* mutant expressing *GATAe* in progenitors using *esg*^*TS*^ for 14 days at 29°C. *esg>GFP* (green) labels midgut progenitors. Each dot represents one gut.

We first observed that over-expressing *GATAe* under the control of *esg*^*TS*^ driver led to the precocious differentiation of progenitors into Pdm1-positive ECs (Fig 8A–8C), consistent with the notion that GATAe can promote progenitor differentiation [33]. The fast differentiation of progenitors was supported by the presence of many mature EC that still kept residual *esg>GFP* signal (Fig 8B), reminiscent of progenitor cells in *esg*^*TS*^*>Sox21a* intestines. Consistent with [33], loss of *GATAe* did not block terminal differentiation in basal conditions, since both Pdm1-positive ECs and Pros-positive EEs were found in stem cell clones deficient for *GATAe* (S8A and S8B Fig). Similarly, EB-specific depletion of *GATAe* using the EB-specific driver *Su(H)GBE*^*TS*^ did not lead to EB tumors, but rather to a slight accumulation of late-stage EBs as judged from their appearance. These results suggest that GATAe may contribute to the rate of EB differentiation. To test this idea, we analyzed the contribution of GATAe to rapid epithelial turnover induced by ingestion of bacteria. Both wild-type flies and flies with EB-specific depletion of *GATAe* were orally infected with *Ecc15* for 2 days, a treatment that increases the pool of progenitors undergoing differentiation, and were further let to recover for 3 days. At this timepoint, the midgut of *Su(H)GBE*^*TS*^*>w* control flies subjected to bacterial challenge had already returned to a homeostatic condition where only nascent EBs with small nuclei were found (Fig 8D). In sharp contrast, accumulation of EBs was observed along the midgut of *Su(H)GBE*^*TS*^*>GATAe-IR* flies (Fig 8E). These EBs had a larger cell size than EBs found in wild-type flies suggesting that they were stuck in the process of differentiation. Consistent with a role of GATAe for accelerated EB differentiation, simultaneously depleting *GATAe* abolished progenitor differentiation in *esg*^*TS*^*>Sox21a* intestines (Fig 4A; S5G Fig). Moreover, expressing *GATAe* with the *esg*^*TS*^ driver suppressed the differentiation defect and tumor formation induced by the loss of either *Sox21a* or *Stat92E* (Fig 8F–8L), revealing that GATAe acts downstream of Sox21a and JAK/STAT. Collectively, our study not only confirms that GATAe contributes to the differentiation process [33], but further reveals its critical role during regeneration as opposed to basal conditions. Our data also show that JAK/STAT-Sox21a-GATAe forms a sequential relay orchestrating the EB-EC differentiation process.

## Discussion

Key questions in stem cell biology are how the pool of stem cells can be robustly expanded yet also timely contracted through differentiation to generate mature cells according to the need of a tissue, and what are the underlying mechanisms that couple stem cell proliferation and differentiation. Over the last years, the mechanisms underlying intestinal stem cell activation have been extensively studied in both flies and mammals [1, 4], while the genetic control of progenitor differentiation, especially during regeneration, has only recently begun to be understood [26, 28, 31].

The transcription factor Sox21a has recently been the focus of studies in fly intestines [31, 32, 45]. Using a *Sox21a-sGFP* transgene, we uncovered its dynamic expression pattern in intestinal progenitors. Higher levels of Sox21a were found in ISC during homeostatic conditions but in EB during regeneration, supporting the roles of Sox21a in both ISC maintenance and EB differentiation at different conditions. The highly dynamic expression pattern of Sox21a revealed by this sGFP-tagged transgene *per se* argues against accumulation and perdurance of GFP fusion protein. Indeed, immunostaining using an antibody against Sox21a also indicated stronger Sox21a expression in ISC in homeostatic condition and global activation of Sox21a in progenitors under DSS-induced regeneration [45]. However, Chen et al., (2016) suggested that Sox21a levels are always higher in EB than in ISC by applying another antibody against Sox21a. The inconsistency between these studies may have arisen from the differences in EB stages examined or the sensitivity of respective detection approaches.

**Fig 9:**
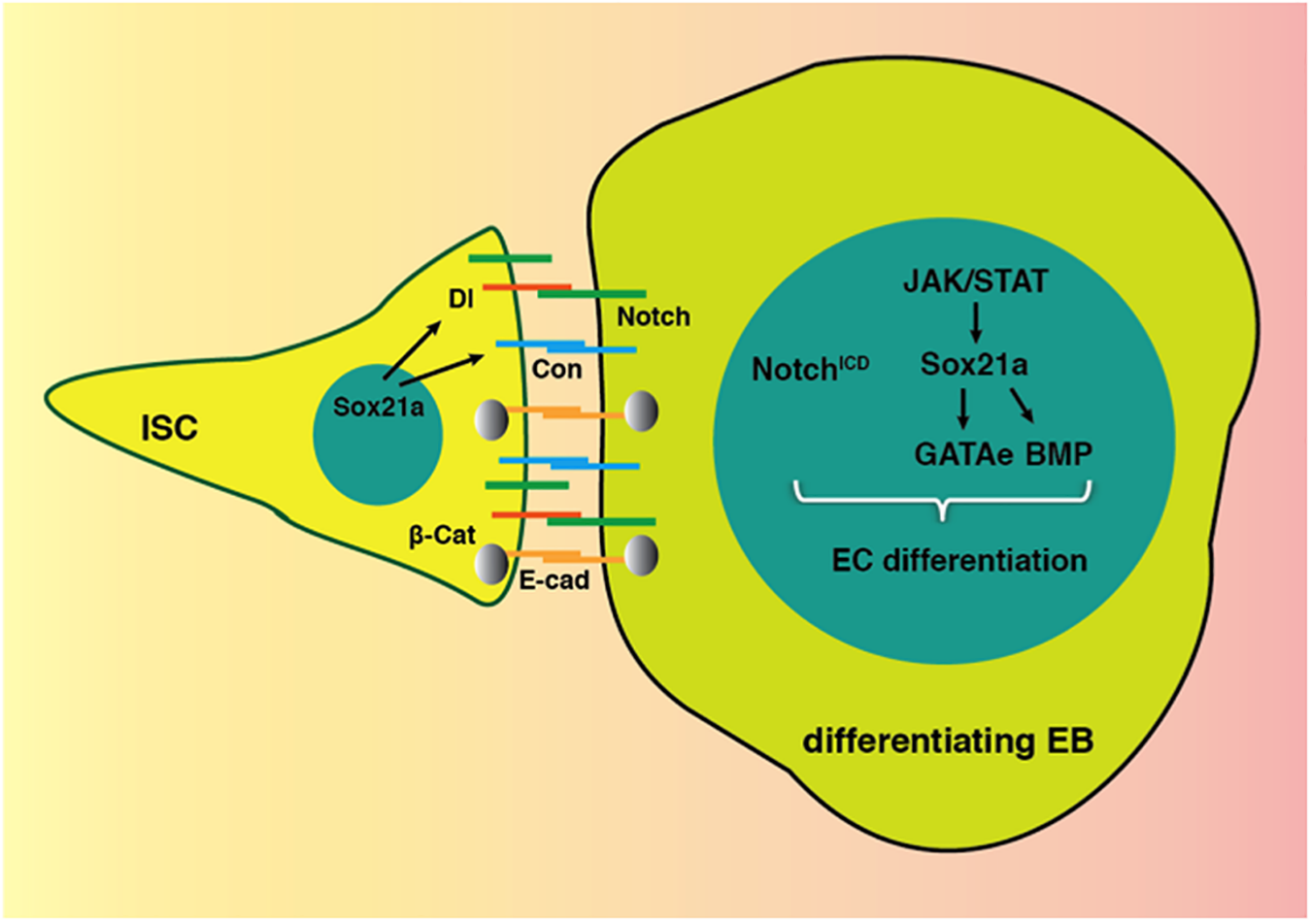
A genetic framework underlies rapid progenitor differentiation for tissue replenishment. During rapid intestinal epithelial renewal, stem cells divide symmetrically with one of the two cells adopting the EB fate. Rapid differentiation involves extended cell contact maintained by the cell adhesion molecules E-cadherin (E-cad) and Connectin (Con) to enhance Notch signaling activity in EBs. A JAK/STAT-Sox21a-GATAe relay further instructs the process of EB to EC differentiation.

In this study, we have analyzed the cellular processes required for efficient progenitor differentiation during regeneration and uncovered three main findings revealing: i) the importance of extended contact between a stem cell and its differentiating daughter, ii) the existence of specific mechanisms allowing fast differentiation during regeneration, and iii) the characterization of a genetic program instructing the transition from EB to EC. These results together led us to propose a molecular framework underlying intestinal regeneration (Fig 9) that is discussed below step by step.

By studying the mechanisms of *Sox21a*-induced differentiation, we found that ISC establishes extended contact with its differentiating daughter within a progenitor pair. Increased interface contact was not only observed upon *Sox21a* expression but also during regeneration after bacterial infection and DSS-feeding. Since the presence of extended contact is rare in intestinal progenitors under homeostatic conditions, we hypothesize that extended contact between progenitors is related to increased epithelial renewal as a mechanism to elicit optimal juxtacrine Notch signaling to accelerate the speed of progenitor differentiation. The observations that down-regulation of the cell adhesion molecules *E-Cadherin* or *Connectin* suppresses rapid progenitor differentiation upon regeneration, and that overexpression of *Connectin* is sufficient to promote differentiation, underline the importance of increased cell-cell contact in rapid differentiation. Our study shows that one early role of Sox21a is to promote the formation of this contact zone, possibly through transcriptional regulation of *Connectin*. Further studies should identify the signals and pathways leading to the change of contact between progenitors to adjust the rate of differentiation.

Intestinal progenitors with extended contact in non-homeostatic midguts have been observed in some studies [14, 41], but their role and significance have not been analyzed. Previous studies have also shown that progenitor nests are outlined by E-Cadherin/β-Catenin complexes [23, 48], yet it was not known whether different degrees of progenitor contact are associated with their ISC versus EB fate. Consistent with our results, recent modeling analyses suggested a positive correlation between the contact area of progenitor pairs and the activation of Notch signaling [53, 54]. Thus, it seems that an increase in the contact area between intestinal progenitors is a hallmark of progenitors that are undergoing accelerated differentiation towards ECs. Another study by Choi et al. (2011) has suggested an inhibitory role of prolonged ISC-EB contact to restrict ISC proliferation. Collectively, these studies and our findings suggest that the strong contact between ISC and EB promotes on one hand the efficient differentiation of EBs into mature intestinal cells while on the other hand preventing stem cells from over-dividing. Thus, we hypothesize that alteration in the contact zone provides a mechanism for ensuring both the appropriate speed of differentiation and the timely resolution of stem cell proliferative capacity.

A second finding of our study consists in revealing the existence of specific mechanisms accelerating differentiation for tissue replenishment. In addition to the extended contact discussed above, we observe a difference in the pattern of ISC division between homeostatic and highly regenerative intestines. The modes of ISC division in *Drosophila* have been the topic of intense discussion, and the general consensus is that it is associated with an asymmetric cell fate outcome, in which one cell remains an ISC and the other engages in differentiation [5, 41, 55, 56]. In line with these previous studies, our results support the notion that asymmetric cell division is the most prevalent mode of ISC division under homeostatic conditions, where the rate of epithelial renewal is low. However, our use of ISC- and EB-specific markers shows that upon rapid regeneration an ISC divides into two cells both expressing the ISC marker Dl-GFP but with one cell showing weak Notch activity. Similarly to other Notch-mediated cell-fate decision systems [57], our study suggests that the two resulting Dl-GFP^+^ cells from a symmetric division stay in close contact and compete for the stem cell fate. While our study is not the first to postulate the existence of symmetric ISC division [5, 55], the use of reliable ISC- and EB-specific markers allows us to better visualize this process. Applying a dual-color lineage tracing system to unravel the final fate of respective cells in a *Dl*^*+*^*—Dl*^*+*^ pair could reinforce the existence of symmetric stem cell division. This is nevertheless technically challenging to apply here since all the current available lineage-tracing settings require a heat shock to initiate the labeling, which affects intestinal homeostasis.

Importantly, we show that the genetic program required for fast intestinal regeneration differs from the one involved in basal intestinal maintenance. Our study indicates that GATAe, Dpp signaling, and the cell adhesion molecules E-cadherin and Connectin are not critical for progenitor differentiation when the rate of epithelial renewal is low, whereas their roles become crucial upon active regeneration. We speculate that many discrepancies in the literature can be reconciled by taking into consideration that some factors are required only for rapid differentiation but not in basal conditions. For instance, the implication of Dpp signaling in differentiation has been disputed, since Zhou et al. (2015) focused on bacterial infection-induced regeneration while the other two studies dealt with basal conditions [34–36]. Our study here points to a clear role of Dpp signaling in the differentiation process upon regeneration. Therefore, better defining the genetic program that allows adjusting the speed of differentiation would be of great interest.

Cell fate determination and differentiation involve extensive changes in gene expression and possibly also gradual change of cell morphology. The EB to EC differentiation in the adult *Drosophila* intestine provides a model of choice to study this process. This transition includes changes in cell shape, an increase in cell size, DNA endoreplication leading to polyploidy and the activation of the set of genes required for EC function (S9A–S9C Fig). In this study, we have integrated a number of pathways (Notch, JAK/STAT and Dpp/BMP) and transcription factors (Sox21a and GATAe) into a sequential framework. We further show that Sox21a contributes to the EB-EC transition downstream of JAK/STAT but upstream of Dpp signaling and GATAe. The recurrent use of several factors, namely JAK/STAT, Sox21a and GATAe at different processes including ISC self-renewal and EB-EC differentiation is likely to be a general feature during cell fate determination, and somehow also complicates the study of differentiation. Future work should analyze how each of the factors interacts with the other in a direct or indirect manner. It would be interesting as well to further study how these factors shape intestinal regionalization as the gut exhibits conspicuous morphological changes along the length of the digestive tract [52, 58].

Several of the findings described are likely to apply to the differentiation program that takes place in mammals. Since Notch signaling plays major roles in stem cell proliferation and cell fate specification from flies to mammals [57, 59], it would be interesting to decipher whether in mammals changes in progenitor contact also impact differentiation speed and whether a specific machinery can accelerate progenitor differentiation when tissue replenishment is required.

## Materials and methods

### *Drosophila* strains

Fly strains were kept on a standard medium (maize flour, dead yeast, agar and fruit juice). *esg-Gal4*, *tub-Gal80*^*TS*^, *UAS-GFP* (referred to as *esg*^*TS*^); *Su(H)GBE-Gal4*, *tub-Gal80*^*TS*^, *UAS-GFP* (referred to as *Su(H)GBE*^*TS*^); *esg-Gal4*, *tub-Gal80*^*TS*^, *UAS-GFP; UAS-Flp*, *Act>>Gal4* (referred to as *esgF/O*); MARCM tester *FRT2A*: *y*,*w*,*hsFlp; tub-Gal4*, *UAS-CD8*::*GFP; FRT2A*, *tub-Gal80;* MARCM tester *FRT82B*: *y*,*w*,*hsFlp*, *tub-Gal4*, *UAS-nlsGFP;;FRT82B*, *tub-Gal80*; *Su(H)-lacZ*, *Sox21a*^*6*^, *UAS-Sox21a*, *UAS-Sox21a-RNAi* (BL53991), *UAS-Stat92E-RNAi* (BL31318 and 35600) and *UAS-N-RNAi* (VDRC100002) have been described before [31]. *Dl-GFP*, *UAS-hep*, *UAS-Raf*^*ACT*^, *UAS-lacZ*, *UAS-N*^*ICD*^, *UAS-arm*^*S10*^, *UAS-Pan*^*DN*^, *UAS-E-cad-RNAi* (BL27689), *UAS-Con-RNAi* (BL28967), *UAS-Dl-RNAi* (BL28032 and 34322), *UAS-arm-RNAi* (BL31304 and 31305), *UAS-Mad-RNAi* (BL31315), *UAS-GATAe-RNAi* (BL 34907), *UAS-Notch-RNAi* (VDRC, KK) and *UAS-mCherry-RNAi* were obtained from Bloomington *Drosophila* Stock Center (BDSC). *Sox21a-GFP* was from VDRC stock center. *UAS-shn*, *UAS-Stat92E* and *UAS-dome*^*DN*^ (gift from Michael Boutros), *FRT82B*, *Neur*^*IF65*^ (gift from Allison Bardin), *Dad-GFP*^*nls*^ (gift from Fisun Hamaratoglu), *UAS-Con* (gift from Rob White) and *FRT82B*, *GATAe*^*1*^ (gift from Takashi Adachi-Yamada) were also used. *UAS-GATAe* was generated in this study.

*Dl-GFP* (BL59819) encodes an endogenously GFP-tagged Dl protein resulting from recombination mediated cassette exchange of a Mi{MIC} insertion in the *Dl* coding intron [38]. This line is homozygous lethal. *Sox21a-GFP* transgenic line derives from a GFP-tagged fosmid clone containing a large genomic region including *Sox21a* [42]. In most cases, the driver lines (*esg*^*TS*^ or *Su(H)GBE*^*TS*^) were crossed to the *w*^*1118*^ strain, *UAS-mCherry-RNAi*, or *UAS-lacZ*, and the progenies were used as control for overexpression experiments. *w*^*1118*^ flies carrying one copy of *esg*^*TS*^ (*esg*^*TS*^*>w*) were used as wild type to visualize the contact between progenitors in different conditions.

### Oral infection with *Ecc15* or other treatments

*Erwinia carotovora carotovora15* (*Ecc15*) was grown in LB medium at 29°C with shaking overnight, and harvested by centrifugation at 3000g at 4°C for 30 minutes. The pellet was then suspended in the residual LB, and bacterial concentration was adjusted to OD_600_ = 200. Flies older than 3 days were first dry-starved in an empty tube for 2 hours, and then transferred into a classical fly food vial containing a filter paper that totally covers the food and was soaked with a solution consisting of 5% sucrose and *Ecc15* at OD200 (1:1), or 6% DSS (average MW 40 kDa, sigma) treated flies were kept at 29°C until dissection. Colcemid treatment was done as reported previously [34]. 200ug/ml colcemid (Sigma) was added to 5% sucrose to pre-treat the flies for 12 hours, and then an *Ecc15* infection was performed in the presence of 200ug/ml colcemid.

### Immunohistochemistry and microscopy

Flies were transferred overnight into a classical fly food vial containing a filter paper soaked with a solution consisting of 5% sucrose to clean the digestive tract. Then, 10–15 intestines of mated adult females were dissected in phosphate-buffered saline (PBS), and fixed for at least one hour at room temperature in 4% paraformaldehyde (PFA) in PBS. They were subsequently rinsed in PBS+0.1% Triton X-100 (PBT), permeabilized and blocked in 2% BSA PBT for one hour, and incubated with primary antibodies in 2% BSA PBT for overnight at 4°C. After one hour of washing, secondary antibodies and DAPI were applied at room temperature for two hours.

Primary antibodies used are: mouse anti-Pros (DSHB, 1:100), mouse anti-Arm (DSHB, 1:100), mouse anti-Dl (DSHB, 1:100), mouse anti-βPS (DSHB, 1:100), mouse anti-Con (DSHB, 1:4), rabbit anti-pH3 (Millipore, 1:1000), Chicken anti-GFP (Abcam, 1:1000), rabbit anti-βGal (Cappel, 1:1000), mouse anti-βGal (Sigma, 1:1000), and Rat anti-mCherry (Life Technologies, 1:500). Alexa488-, Alexa555- or Alexa647-conjugated secondary antibodies (Life Technologies) were used. Nuclei were counterstained by DAPI (Sigma, 1:10’000). All the images were taken on a Zeiss LSM 700 confocal microscope at BIOP in EPFL. Images were processed using Image J and Adobe Photoshop software. Shown in figures are maximal intensity projections of all the confocal z stacks.

### Generation of *UAS-GATAe* transgene

To generate *the UAS-GATAe* construct, the following primers (*caccATGGTCTGCAAAACTATCTC* and *TTAGTTATTCGATGATCGCTC*) were used to amplify the 2.2kb *GATAe*-PA coding regions from cDNA clone LD08432 purchased from DGRC. The PCR product was first cloned into pENTR-D-TOPO (Life Technologies) vector, and then swapped into pTW destination vector to make *UAS-GATAe*. Transgenic flies were established by standard *P* element-mediated germ-line transformation (BestGene Inc.). At least three independent transgenic lines were tested for expression level.

### Conditional expression of UAS-linked transgenes

The TARGET system was used in combination with the indicated Gal4 drivers to conditionally express UAS-linked transgenes [60]. Flies were grown at 18°C to limit Gal4 activity. After 3–5 days at 18°C, adult flies with the appropriate genotypes were shifted to 29°C, a temperature inactivating the temperature-sensitive Gal80’s ability to suppress Gal4, and dissected after indicated time of transgene activation.

### MARCM clone induction

Mosaic analysis with a repressible cell marker (MARCM) technique was used for clonal analysis [61]. For clone induction, 3-5-day-old flies with the appropriate genotypes were heat-shocked for 30 min at 37.5°C in a water bath. The flies were immediately transferred into a new tube and kept at 25°C or indicated temperature until dissection. Overexpression experiments were performed by combining the UAS-linked transgenes with the *FRT2A*, the *FRT2A*, *Sox21a*^*6*^, or the *FRT82B*, *Neur*^*IF65*^ chromosome. Note that UAS-linked transgenes were only expressed in the clones indicated by the presence of GFP.

### FACS for RNA-seq and RNA-seq data analysis

*esg*^*TS*^ virgin females were crossed to either *w*^*1118*^ as control or *UAS-Sox21a* for overexresspion at 18°C. Eclosed flies were maintained at 18°C for 5–7 days. Around 50 flies for each biological replicate were dissected in ice-cold 1xPBS made with DEPC-treated water under a dry-ice chilled dissecting microscope, within a one-hour time frame. Proventriculus, hindgut and midgut/hindgut junction were removed to collect only midgut *esg>GFP* positive cells. Two biological replicates were performed, and the activation of *Sox21a* expression was done by shifting *esg*^*TS*^*>Sox21a* flies to 29°C for 12 hours and 24 hours, respectively. Cell dissociation, FACS sorting, total RNA isolation and mRNA amplification were performed as described [31]. RNA-seq was performed on a Hi-Seq2000 (Illumina) with 100 nt single-end sequencing, and sequencing data was analyzed as described before [31]. Sequencing data will be deposited in public database prior to publication.

### qRT-PCR analysis of gene expression

Total RNA was extracted from dissected whole guts (12–15 guts per sample) using Trizol and cDNA was synthesized using the PrimeScript RT reagent Kit (TaKaRa). 0.5μg total RNA was used for reverse transcription with oligo dT, and the 1^st^ strand cDNA was diluted 10–20 times with water to be further used in real time PCR. Real time PCR was performed in triplicate for each sample using SYBR Green (Roche) on a LightCycler 480 System (Roche). Expression values were calculated using the ΔΔCt method and relative expression was normalized to *Act5C*. Results are shown as mean ± SEM of at least 3 independent biological samples. Statistical analysis was performed in Prism Software using the unpaired t test. Primers used for qPCR are as follows.

*Dl*: *5’TGTGAACATGGACATTGCGA3’* and *5’GTCTGTGGTTGGTGCAGTAG3’*
*Sox21a*: *5’GGACAGAAGCGTCCATTCAT3’* and *5’TGACTTGTTGAGCGTCTTGG3’*
*Act5C*: *5’CAGAGCAAGCGTGGTATCCT3’* and *5’GGTGTGGTGCCAGATCTTCT3’*

### Statistical analysis

All analyses were done with GraphPad Prism. Unpaired t test were used unless otherwise noted. *p* values are indicated by *p < 0.05, **p < 0.01, ***p < 0.001, ns: p > 0.05. Shown are means and SEM. Data are representative of at least three experiments. Each dot represents one gut except Fig 5E. Sample size is also indicated in the figures.

## Supporting information

S1 Fig Increased progenitor contact is a general feature of regenerating intestine. **(A)** Representative intestine of flies orally treated with 6% DSS for 20 hours. Note the presence of progenitor pairs with strong cell-cell contact as revealed by Arm staining (indicated by orange arrows). Pros (red, nuclei) marks EE. **(B-C)** Representative intestines of flies orally treated with 200ug/ml colcemid for 12 hours and then further challenged with *Ecc15* for 10hours (C) and unchallenged control (B). Note that colcemid feeding arrests stem cells in metaphase and this treatment inhibits the formation of increased cell junction that is normally induced by *Ecc15* infection. Cells in metaphase were marked with an antibody against Phospho-Histone H3 (PH3, a mitotic marker, in white). **(D-E)** Two representative images of midgut from *Sox21a*^*6*^ mutant flies orally infected with *Ecc15* for 10 hours. Long junctions between progenitors (indicated with orange arrows and marked by Arm staining) form normally. **(F)** Representative image of midgut from *esg*^*TS*^*>Notch-IR* flies shifted to 29°C for 4 days to induce Notch tumor formation. Note the presence of long junctions (shown with *esg>mCD8*::*GFP*) between ISCs within each cluster.
(TIF)

S2 Fig Bacterial infection alters the mode of ISC division. (**A-G**) Representative images of progenitor cells from intestines of flies orally infected with *Ecc15*. ISCs are marked with *Dl-GFP* (green) and EBs with *Su(H)-lacZ* (red). Other markers (βPS, Dl, Prospero or PH3) are shown in gray. βPS (beta-integrin) highlights the basal extracellular matrix, Prospero marks EEs and PH3 is a mitotic marker. Images in (A-B) are sagittal views of the intestinal epithelium and others (C-G) show frontal plane. Note that the two cells in the ISC pair in (A) are both basally localized, but one of the two ISCs expresses weak Notch reporter (red). In comparison, progenitors with strong Notch activity exhibit more apical localization (B). A mitotic cell is shown in (E). (F and G) show ISC pairs that have just derived from an ISC division, and one cell within the ISC-ISC pair in (G) expresses weak *Su(H)-lacZ*.
(TIF)

S3 Fig Sox21a regulates intestinal regeneration. **(A-D**) *Sox21a* expression in control flies (A) and flies over-expressing *hep* (*hemipterous*, encoding the JNK kinase), B) or *Raf*^*ACT*^ (C-D) in intestinal progenitors using *esg*^*TS*^ for 36 hours. *Sox21a* expression is monitored by the *Sox21a-GFP* transgene (green). Mitotic cells were marked with an antibody against Phospho-Histone H3 (PH3, a mitotic marker, in red). Note that the higher levels of *Sox21a* reporter expression are found in nuclei of large size (indicated with yellow arrows in B-D) within progenitor cells, presumably differentiating EBs. Strikingly, expression of *Raf*^*ACT*^ generated giant progenitors with nuclei of larger size than that of surrounding enterocytes, in line with the potent growth-promoting function of Ras/MAPK signaling. (**E**) Quantification of mitotic index in the midgut of flies with the indicated genotype. (**F**) Sagittal view of the midgut progenitors expressing *Sox21a* for 36 hours. Progenitors (marked by *esg>GFP*), E-Cadherin junction (marked by Arm) and nuclei (DAPI) are shown. In each case, *esg>GFP*^*weak*^ cells maintain a strong contact with another more basally localized *esg>GFP*^*strong*^ cell, as revealed by increased Arm staining in the junction. Each dot represents one gut.
(TIF)

S4 Fig Sox21a activates *Dl* transcription. qPCR measurement of mRNA levels of *Sox21a* and *Dl* in dissected midgut of flies with indicated genotypes after activation of transgene expression for 36 hours. Expression is normalized to *Act5C*.
(TIF)

S5 Fig Sox21a-induced differentiation requires multiple factors. (**A-I**) Representative intestines of *esg*^*TS*^*>Sox21a* flies co-expressing various transgenes (as indicated) for 36 hours. Progenitor cells are shown separately on the right panel (revealed by *esg>GFP*). Left panel shows the merge of DAPI (blue), *esg>GFP* (green) and Arm (red) channels. Prospero (EE marker) is also shown in a subset of images (A, B, D, F, G and I). Full quantification of the differentiation phenotype is shown in Fig 4A.
(TIF)

S6 Fig Regulation of progenitor differentiation by cell adhesion molecules. **(A)** Immunostaining of midgut progenitors overexpressing Connectin (*esg*^*TS*^*>Connectin*) with an anti-Connectin antibody. Note that Connectin is localized to the membrane junctions between progenitors. (**B-C**) Control intestine (B) and progenitor-specific expression of *Connectin* (C) using *esg*^*TS*^ for 4 days. Note the absence of EEs (Pros^+^) from the region with big cluster of *esg>GFP*^+^ cells. *esg>GFP* (in green), Arm (in red, membrane), Pros (in red, nuclei, indicated with yellow arrows) and DAPI staining are shown. **(D-G)** Midguts of flies with indicated genotype shifted to 29°C for 4 days and then either challenged with *Ecc15* for 12hours (F-G) or unchallenged (UN, D-E). Orange arrows indicate differentiating EBs. Mitotic cells are marked with PH3 in red. (**H**) Quantification of mitotic index in the midgut of flies with the indicated genotype 4 days after transgene expression. Each dot represents one gut. **(I-J)** Midgut turnover revealed by the *esgF/O* system with control (I) or *Connectin* (J) overexpression for 7 days at 29°C. Note that the number of Pros (in red, nuclei, indicated with yellow arrows) expressing cells is largely reduced in (J). (**K-M**) basal-level midgut turnover revealed by the *esgF/O* system with control (K), *E-cad* (L) or *Connectin* (M) knockdown for 2 weeks at 29°C.
(TIF)

S7 Fig Sox21a-induced differentiation requires functional Notch signaling. (**A-B**) Images of intestines from fly co-expressing *Sox21a* and *Notch-RNAi* using *esg*^*TS*^ for 36 hours. Progenitors are shown with both *esg>GFP* and Arm. Likely due to the relative efficiency between *Sox21a* overexpression and *Notch* depletion, half number of the intestines (n = 28) develop ISC tumors (as shown in A), and half possess both small ISC tumors and differentiating EBs (as shown in B). (**C-D**) A *Neur*^*IF65*^ mutant MARCM clone (C) and a *Neur*^*IF65*^ mutant clone co-expressing *Sox21a* (D) are analyzed 14 days after clone induction. *Neuralized* (*Neur*) encodes for an E3 ubiquitin ligase that is essential for Notch signaling. Note that ISC tumors produced in *Neur*^*IF65*^ mutant clones are not suppressed by the co-expression of *Sox21a*, indicating that functional Notch signaling is a prerequisite for *Sox21a*-induced EC differentiation.
(TIF)

S8 Fig *GATAe* mutation does not block intestinal progenitor differentiation under basal condition. (**A-B**) Wild type MARCM clones (positively labeled by GFP, A) and clones mutant for *GATAe* (B) are analyzed 4 days after clone induction. EEs express Prospero (nuclei), and ECs are marked by Pdm1. Note that EC or EE differentiation was not blocked in *GATAe* mutant clone.
(TIF)

S9 Fig EB to EC differentiation is a multistep process. (**A-B**) Representative images of control (A) and *Ecc15*-infected (B) intestines. EBs are visualized by the expression of *Su(H)GBE>GFP* (green). Nuclei are stained by DAPI. (**C**) Representative EBs in the course of maturation toward EC, redrawn from (B). In the absence of challenge, nascent EBs from 5–7 day-old adults exhibits a small size (A). Oral ingestion of *Ecc15* causes damage to the intestinal epithelium and quickly triggers ISC activity for regeneration. Under this condition, all the intermediate states between a nascent EB and a mature EC become detectable (C).
(TIF)

S1 Table RNA-seq data. The datasets were generated from FACS-sorted progenitors of flies overexpressing *Sox21a* with *esg*^*TS*^ for 12 or 24 hours, and compared to control (*esg*^*TS*^*>w*). Only differentially expressed genes (12h vs Control, and 24h vs Control) with *p* value less than 0.05 are listed. Expression value, fold change, *p* value, gene name and GO terms are included.
(XLS)
