# Supplementary figures and images for: A genetic framework controlling the differentiation of intestinal stem cells during regeneration in Drosophila
Source: PLoS Genet. 2017 Jun 29;13(6):e1006854. doi: 10.1371/journal.pgen.1006854 (PMC5510897; doi:10.1371/journal.pgen.1006854)

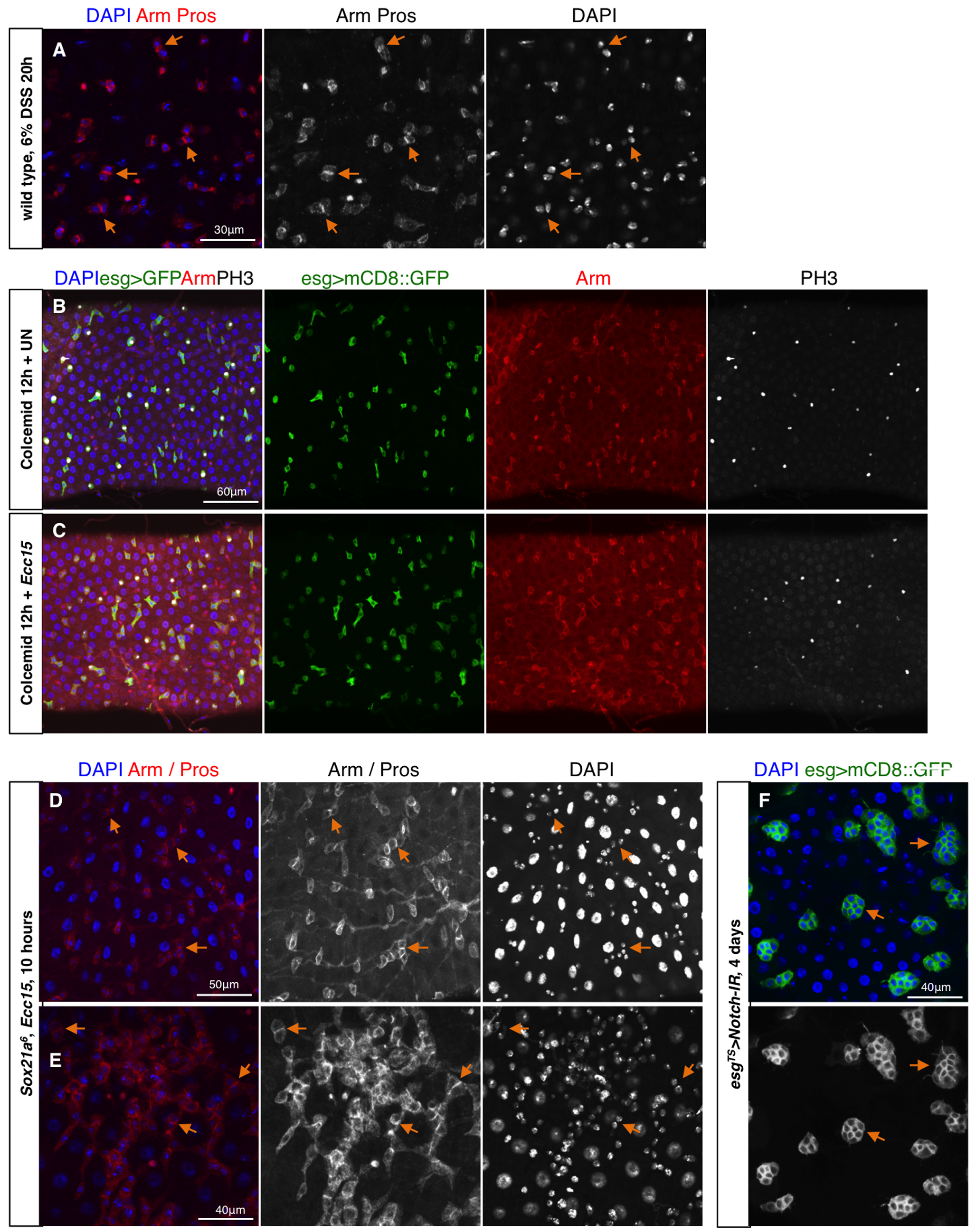

Supplement: S1 Fig — (A) Representative intestine of flies orally treated with 6% DSS for 20 hours. Note the presence of progenitor pairs with strong cell-cell contact as revealed by Arm staining (indicated by orange arrows). Pros (red, nuclei) marks EE. (B-C) Representative intestines of flies orally treated with 200ug/ml colcemid for 12 hours and then further challenged with Ecc15 for 10hours (C) and unchallenged control (B). Note that colcemid feeding arrests stem cells in metaphase and this treatment inhibits the formation of increased cell junction that is normally induced by Ecc15 infection. Cells in metaphase were marked with an antibody against Phospho-Histone H3 (PH3, a mitotic marker, in white). (D-E) Two representative images of midgut from Sox21a6 mutant flies orally infected with Ecc15 for 10 hours. Long junctions between progenitors (indicated with orange arrows and marked by Arm staining) form normally. (F) Representative image of midgut from esgTS>Notch-IR flies shifted to 29°C for 4 days to induce Notch tumor formation. Note the presence of long junctions (shown with esg>mCD8::GFP) between ISCs within each cluster. (TIF) [file pgen.1006854.s001.tif]

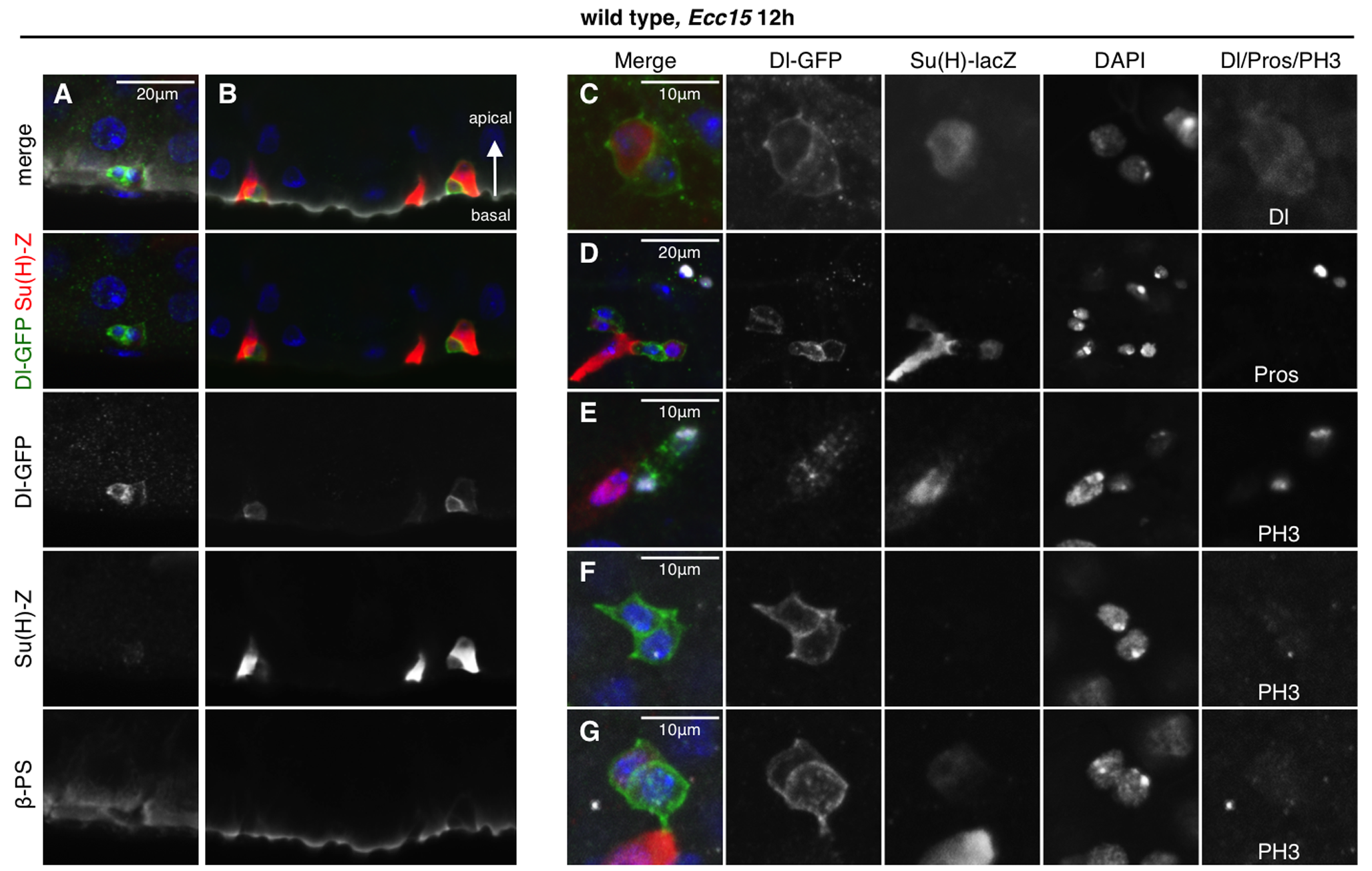

Supplement: S2 Fig — (A-G) Representative images of progenitor cells from intestines of flies orally infected with Ecc15. ISCs are marked with Dl-GFP (green) and EBs with Su(H)-lacZ (red). Other markers (βPS, Dl, Prospero or PH3) are shown in gray. βPS (beta-integrin) highlights the basal extracellular matrix, Prospero marks EEs and PH3 is a mitotic marker. Images in (A-B) are sagittal views of the intestinal epithelium and others (C-G) show frontal plane. Note that the two cells in the ISC pair in (A) are both basally localized, but one of the two ISCs expresses weak Notch reporter (red). In comparison, progenitors with strong Notch activity exhibit more apical localization (B). A mitotic cell is shown in (E). (F and G) show ISC pairs that have just derived from an ISC division, and one cell within the ISC-ISC pair in (G) expresses weak Su(H)-lacZ. (TIF) [file pgen.1006854.s002.tif]

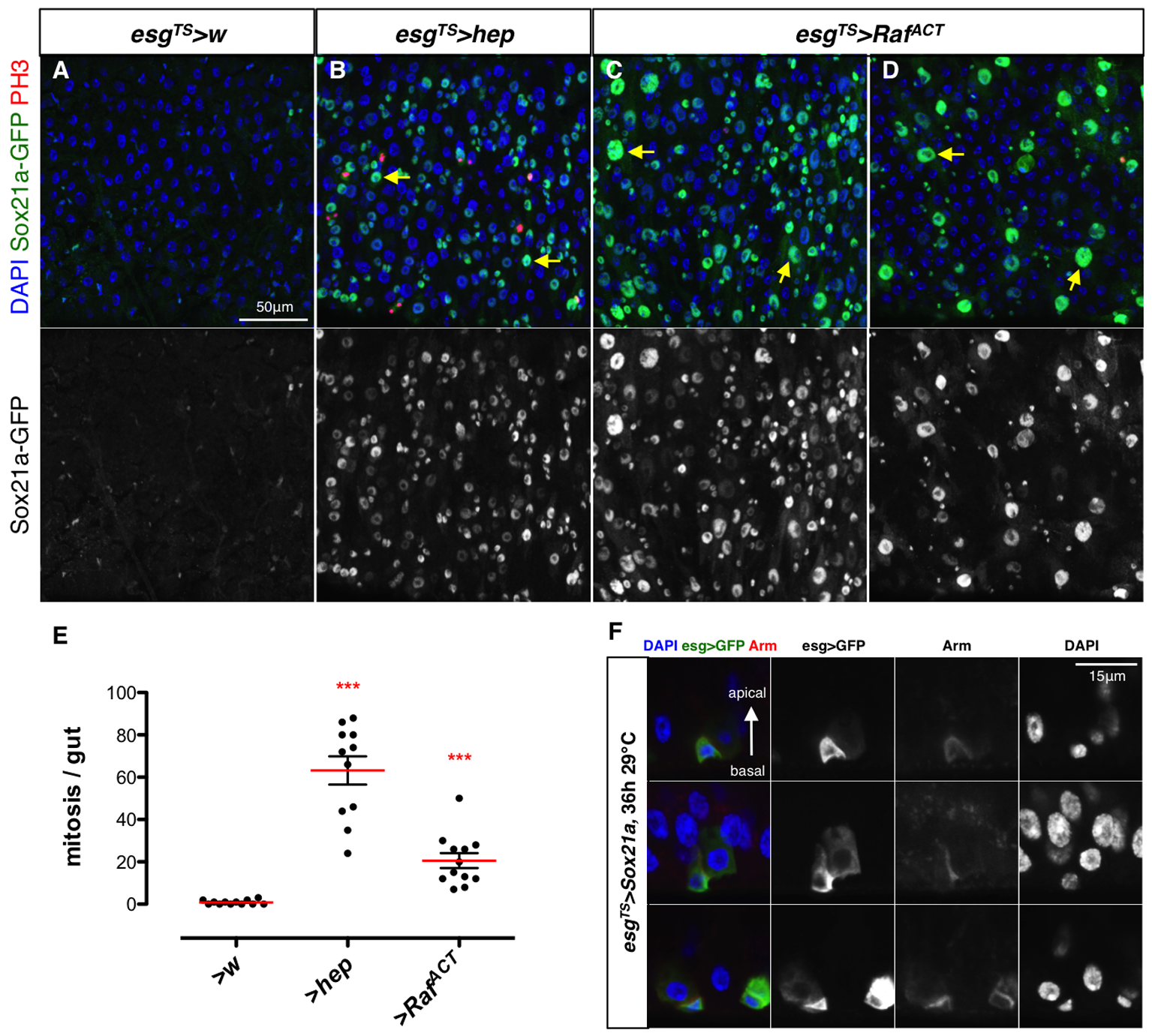

Supplement: S3 Fig — (A-D) Sox21a expression in control flies (A) and flies over-expressing hep (hemipterous, encoding the JNK kinase), B) or RafACT (C-D) in intestinal progenitors using esgTS for 36 hours. Sox21a expression is monitored by the Sox21a-GFP transgene (green). Mitotic cells were marked with an antibody against Phospho-Histone H3 (PH3, a mitotic marker, in red). Note that the higher levels of Sox21a reporter expression are found in nuclei of large size (indicated with yellow arrows in B-D) within progenitor cells, presumably differentiating EBs. Strikingly, expression of RafACT generated giant progenitors with nuclei of larger size than that of surrounding enterocytes, in line with the potent growth-promoting function of Ras/MAPK signaling. (E) Quantification of mitotic index in the midgut of flies with the indicated genotype. (F) Sagittal view of the midgut progenitors expressing Sox21a for 36 hours. Progenitors (marked by esg>GFP), E-Cadherin junction (marked by Arm) and nuclei (DAPI) are shown. In each case, esg>GFPweak cells maintain a strong contact with another more basally localized esg>GFPstrong cell, as revealed by increased Arm staining in the junction. Each dot represents one gut. (TIF) [file pgen.1006854.s003.tif]

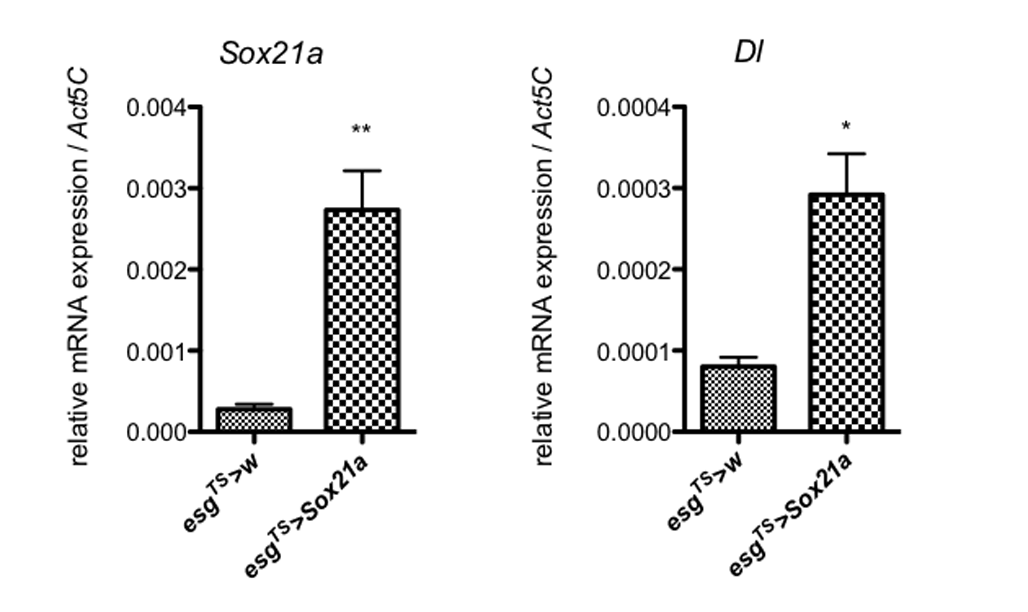

Supplement: S4 Fig — qPCR measurement of mRNA levels of Sox21a and Dl in dissected midgut of flies with indicated genotypes after activation of transgene expression for 36 hours. Expression is normalized to Act5C. (TIF) [file pgen.1006854.s004.tif]

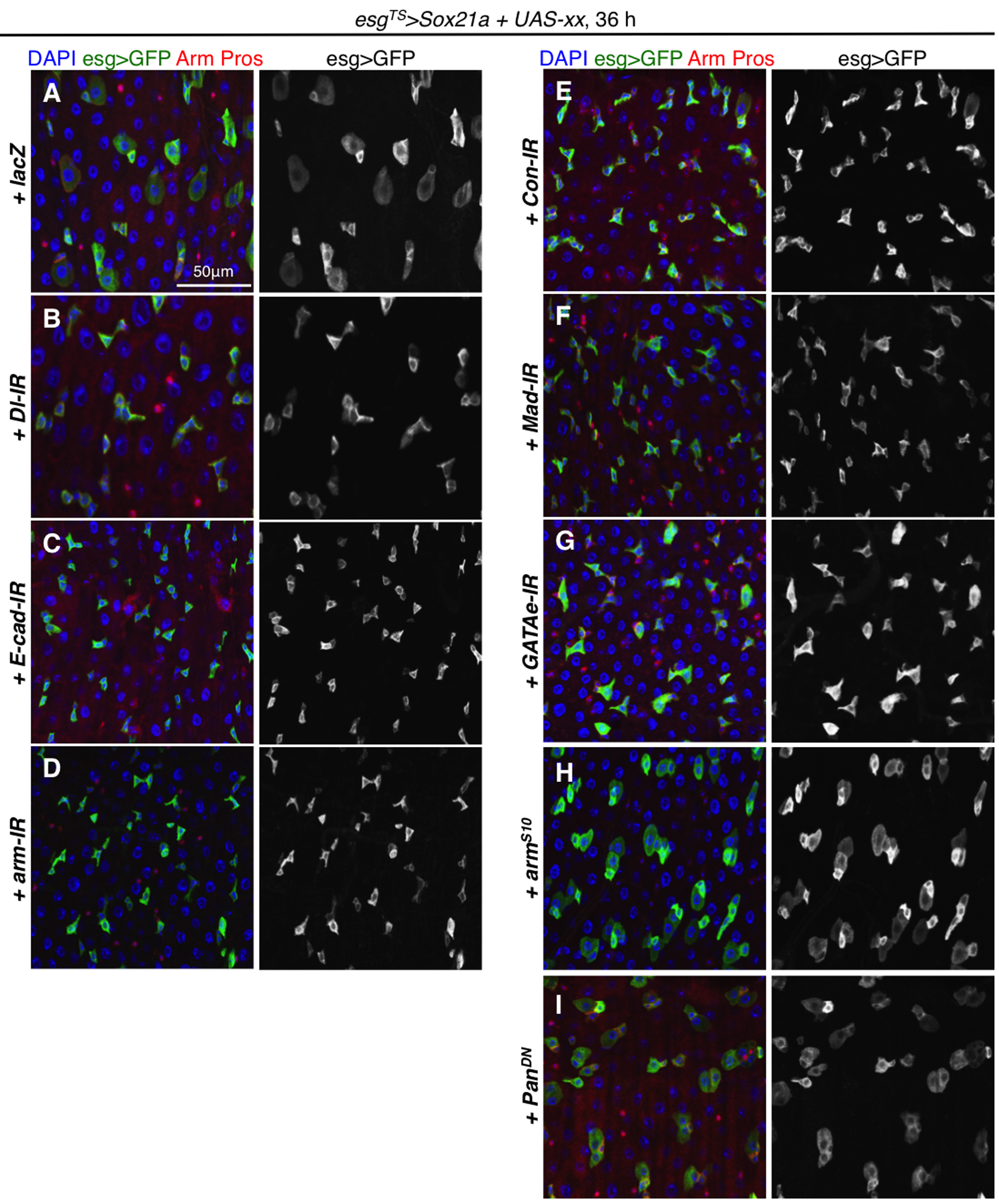

Supplement: S5 Fig — (A-I) Representative intestines of esgTS>Sox21a flies co-expressing various transgenes (as indicated) for 36 hours. Progenitor cells are shown separately on the right panel (revealed by esg>GFP). Left panel shows the merge of DAPI (blue), esg>GFP (green) and Arm (red) channels. Prospero (EE marker) is also shown in a subset of images (A, B, D, F, G and I). Full quantification of the differentiation phenotype is shown in Fig 4A. (TIF) [file pgen.1006854.s005.tif]

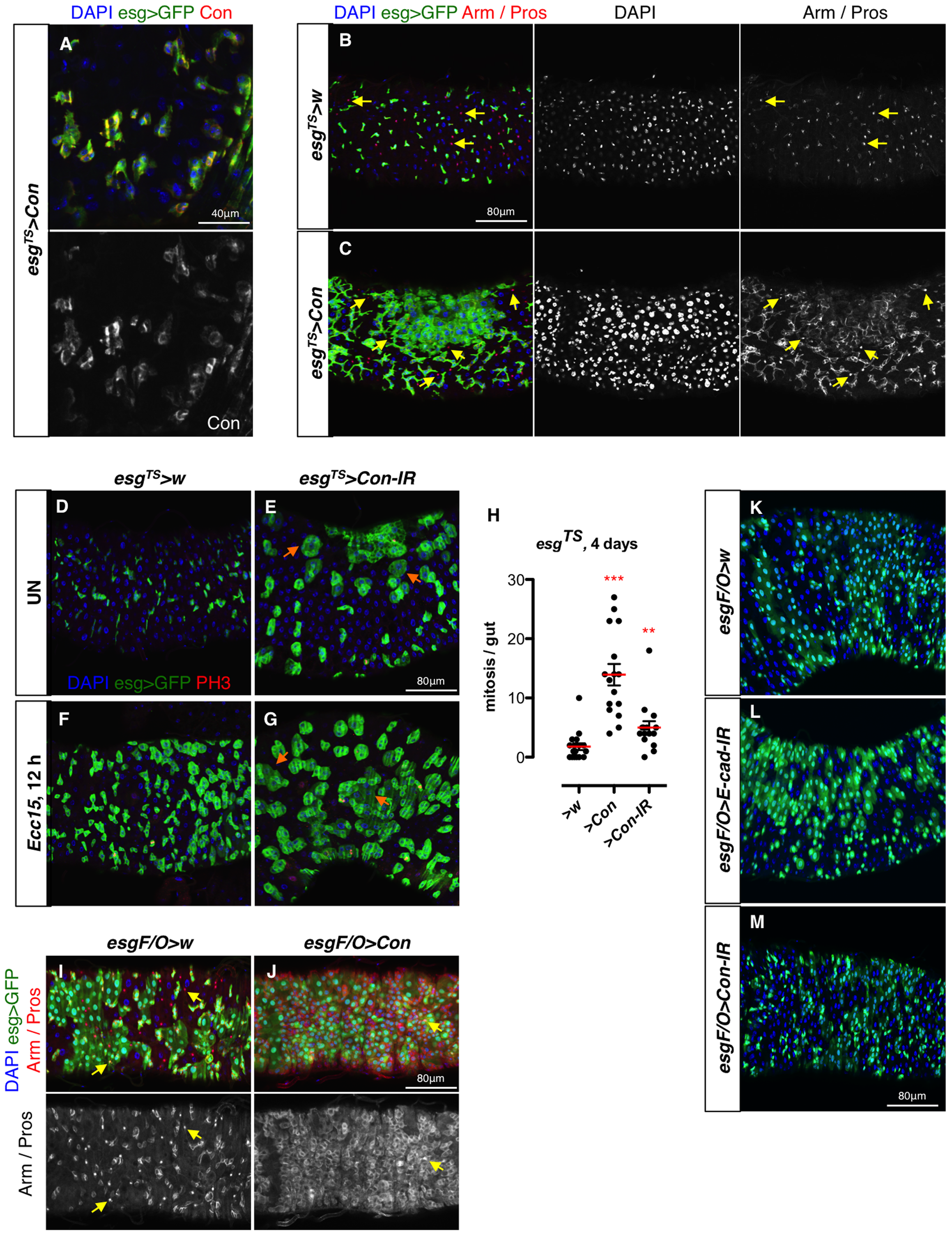

Supplement: S6 Fig — (A) Immunostaining of midgut progenitors overexpressing Connectin (esgTS>Connectin) with an anti-Connectin antibody. Note that Connectin is localized to the membrane junctions between progenitors. (B-C) Control intestine (B) and progenitor-specific expression of Connectin (C) using esgTS for 4 days. Note the absence of EEs (Pros+) from the region with big cluster of esg>GFP+ cells. esg>GFP (in green), Arm (in red, membrane), Pros (in red, nuclei, indicated with yellow arrows) and DAPI staining are shown. (D-G) Midguts of flies with indicated genotype shifted to 29°C for 4 days and then either challenged with Ecc15 for 12hours (F-G) or unchallenged (UN, D-E). Orange arrows indicate differentiating EBs. Mitotic cells are marked with PH3 in red. (H) Quantification of mitotic index in the midgut of flies with the indicated genotype 4 days after transgene expression. Each dot represents one gut. (I-J) Midgut turnover revealed by the esgF/O system with control (I) or Connectin (J) overexpression for 7 days at 29°C. Note that the number of Pros (in red, nuclei, indicated with yellow arrows) expressing cells is largely reduced in (J). (K-M) basal-level midgut turnover revealed by the esgF/O system with control (K), E-cad (L) or Connectin (M) knockdown for 2 weeks at 29°C. (TIF) [file pgen.1006854.s006.tif]

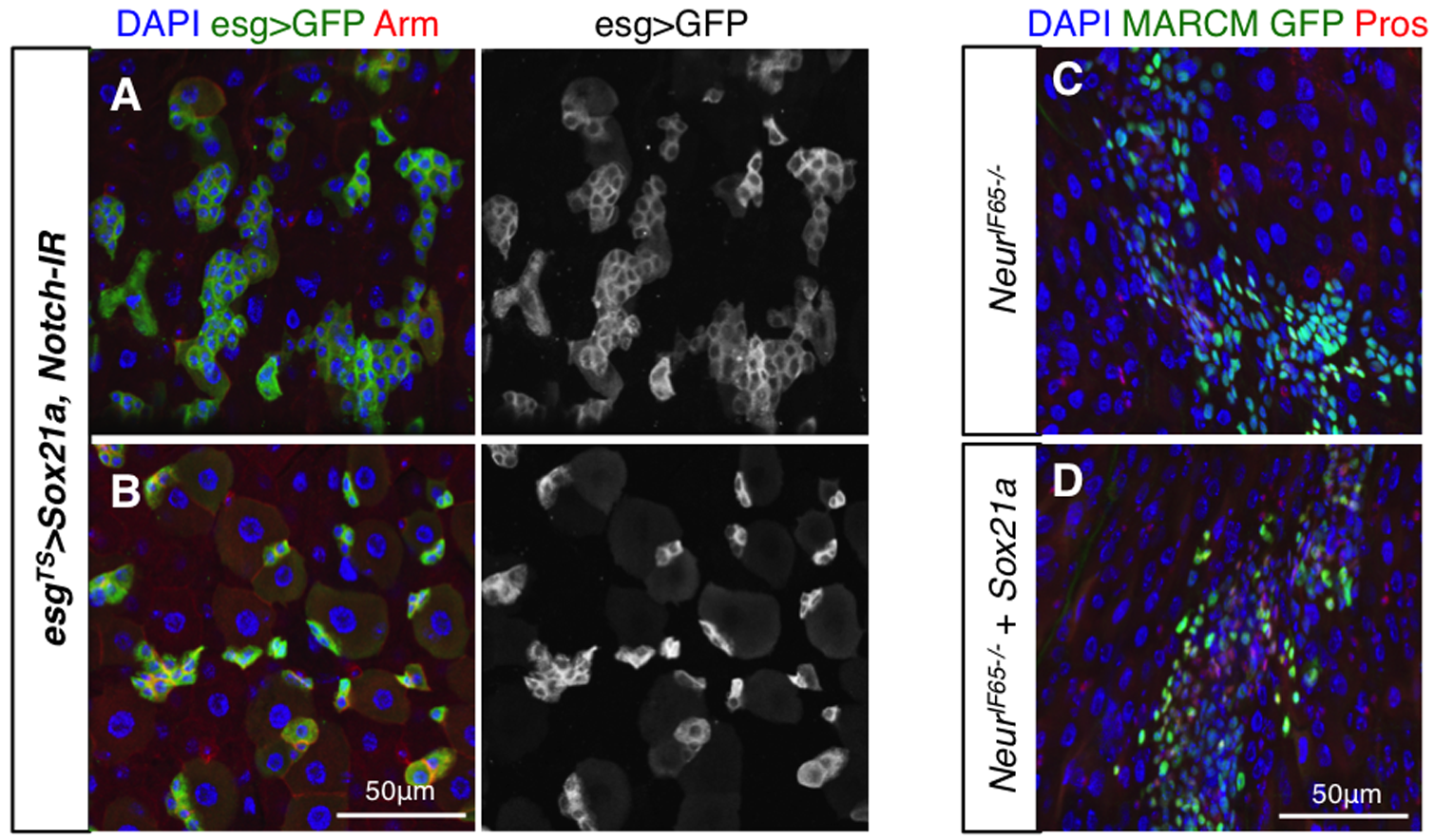

Supplement: S7 Fig — (A-B) Images of intestines from fly co-expressing Sox21a and Notch-RNAi using esgTS for 36 hours. Progenitors are shown with both esg>GFP and Arm. Likely due to the relative efficiency between Sox21a overexpression and Notch depletion, half number of the intestines (n = 28) develop ISC tumors (as shown in A), and half possess both small ISC tumors and differentiating EBs (as shown in B). (C-D) A NeurIF65 mutant MARCM clone (C) and a NeurIF65 mutant clone co-expressing Sox21a (D) are analyzed 14 days after clone induction. Neuralized (Neur) encodes for an E3 ubiquitin ligase that is essential for Notch signaling. Note that ISC tumors produced in NeurIF65 mutant clones are not suppressed by the co-expression of Sox21a, indicating that functional Notch signaling is a prerequisite for Sox21a-induced EC differentiation. (TIF) [file pgen.1006854.s007.tif]

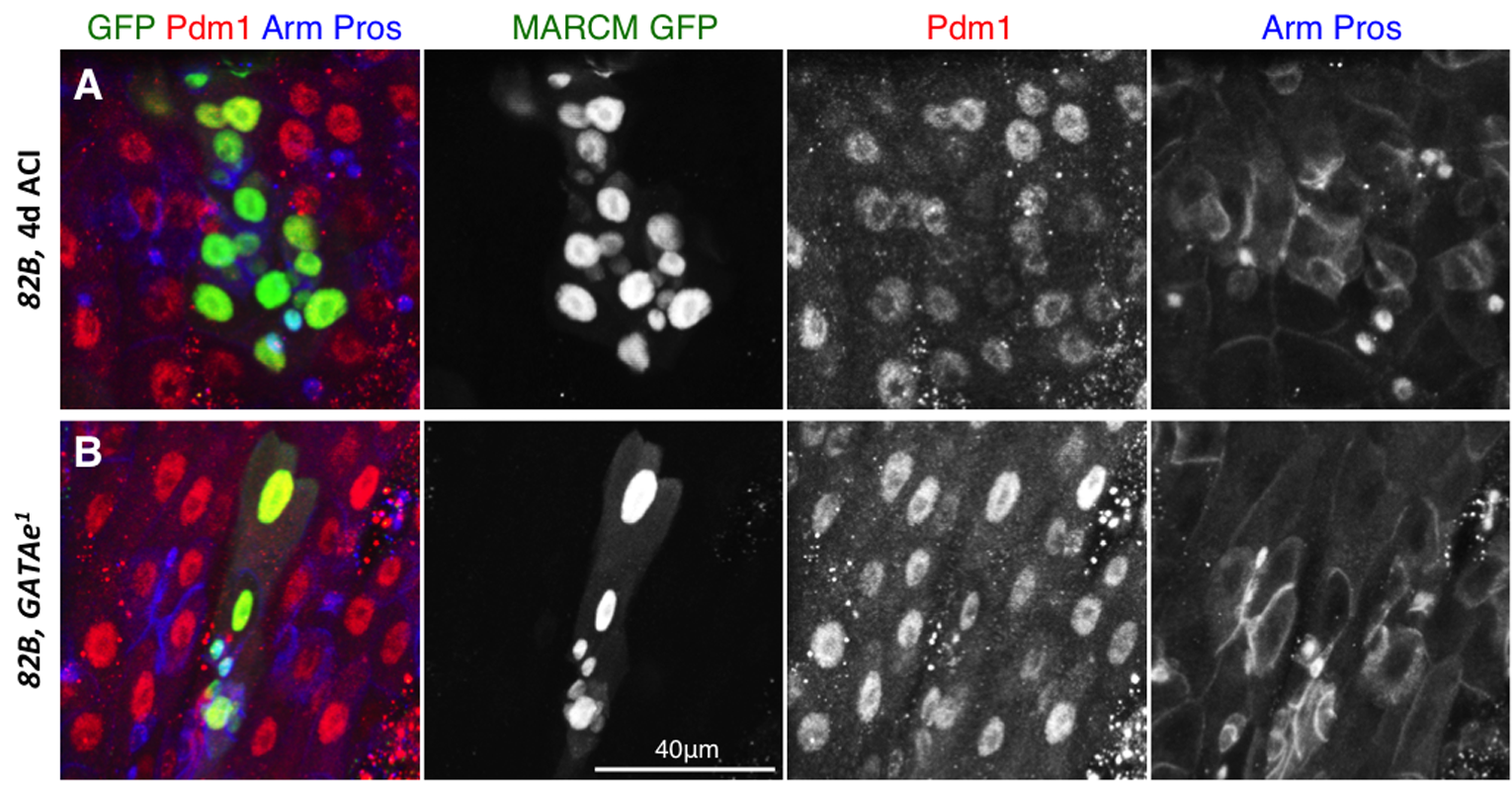

Supplement: S8 Fig — (A-B) Wild type MARCM clones (positively labeled by GFP, A) and clones mutant for GATAe (B) are analyzed 4 days after clone induction. EEs express Prospero (nuclei), and ECs are marked by Pdm1. Note that EC or EE differentiation was not blocked in GATAe mutant clone. (TIF) [file pgen.1006854.s008.tif]

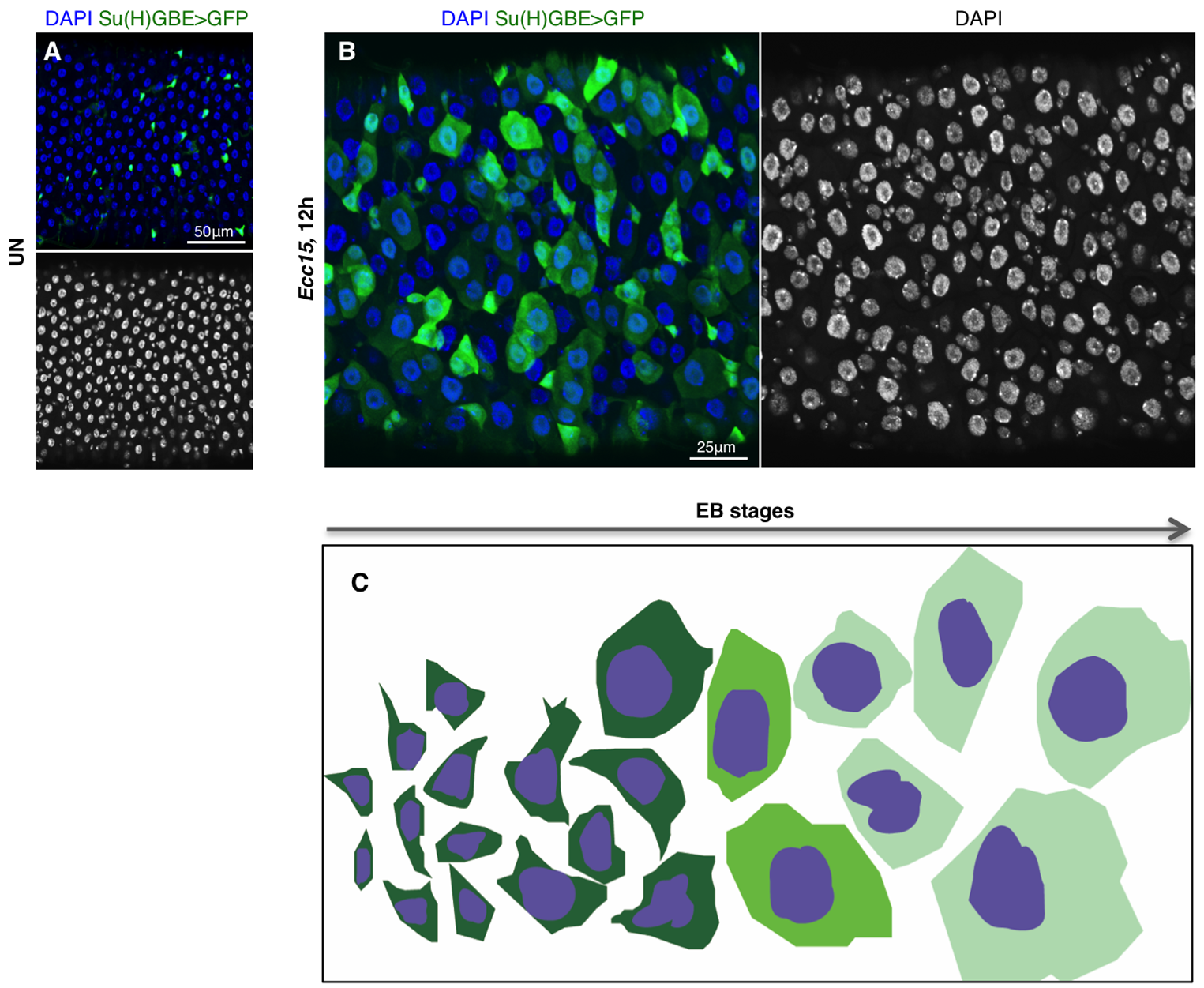

Supplement: S9 Fig — (A-B) Representative images of control (A) and Ecc15-infected (B) intestines. EBs are visualized by the expression of Su(H)GBE>GFP (green). Nuclei are stained by DAPI. (C) Representative EBs in the course of maturation toward EC, redrawn from (B). In the absence of challenge, nascent EBs from 5–7 day-old adults exhibits a small size (A). Oral ingestion of Ecc15 causes damage to the intestinal epithelium and quickly triggers ISC activity for regeneration. Under this condition, all the intermediate states between a nascent EB and a mature EC become detectable (C). (TIF) [file pgen.1006854.s009.tif]
